# Advances in Immunosuppressive Agents Based on Signal Pathway

**DOI:** 10.3389/fphar.2022.917162

**Published:** 2022-05-26

**Authors:** Zhiqing Xu, Ming Chu

**Affiliations:** ^1^ Department of Immunology, National Health Commission (NHC) Key Laboratory of Medical Immunology (Peking University), School of Basic Medical Sciences, Peking University, Beijing, China; ^2^ Department of Pharmacology, Jilin University, Changchun, China

**Keywords:** immunosuppressive agent, jak-stat, NF-κB, PI3K-AKT-mTOR, MAPK, Keap1/Nrf2-ARE

## Abstract

Immune abnormality involves in various diseases, such as infection, allergic diseases, autoimmune diseases, as well as transplantation. Several signal pathways have been demonstrated to play a central role in the immune response, including JAK/STAT, NF-κB, PI3K/AKT-mTOR, MAPK, and Keap1/Nrf2/ARE pathway, in which multiple targets have been used to develop immunosuppressive agents. In recent years, varieties of immunosuppressive agents have been approved for clinical use, such as the JAK inhibitor tofacitinib and the mTOR inhibitor everolimus, which have shown good therapeutic effects. Additionally, many immunosuppressive agents are still in clinical trials or preclinical studies. In this review, we classified the immunosuppressive agents according to the immunopharmacological mechanisms, and summarized the phase of immunosuppressive agents.

## Introduction

Immunosuppressive agents are a class of drugs that inhibit the abnormal immune response of the body and suppress the proliferation and function of cells related to the immune response (macrophages such as T cells and B cells), thereby reducing the antibody immune response, and are now mainly used in organ transplantation anti-rejection reactions and autoimmune diseases. Undoubtedly, immunosuppressive agents have achieved great progress and success in the treatment of these diseases, thus further demonstrating the great research and development prospects of immunosuppressive agents. In 1949, Edward and Philip successfully extracted the adrenocorticotropic hormone cortisone from animals and elucidated its structure and biological effects. Since then, immunosuppressive agents have been on the stage of history, and glucocorticoids have been widely used in clinical practice, and two great scientists were awarded the Nobel Prize in 1950 for this discovery. However, medical problems abound, and the quest of our ancestors never stops. In the 1950s, the anti-rejection reactions present in organ transplants could only be controlled by radiation, and patient survival could not be guaranteed. Subsequently, medical personnel applied glucocorticoids to organ transplants, but the quality of patient survival was delayed due to the highly toxic side effects of hormones, and the development of immunosuppressive agents was imminent. It was not until 1953 when George Hitchings and his associate Gertrude Elion successfully developed the anti-cancer drug 6-mercaptopurine and structurally modified it to develop mercaptopurine, which was used in combination with hormonal drugs for organ transplantation, that the life span of transplanted organs was greatly extended, but research into the use of new drugs for organ transplantation is still progressing at a rapid pace today. Although the problems of organ transplantation have improved, new problems have arisen. We all know that the use of immunosuppressive drugs is a very effective way to treat autoimmune diseases, but the drugs used need constant innovation. In the early days, autoimmune diseases were mainly treated with glucocorticoids and cytotoxic drugs, but it was found that these two types of drugs were too selective for cells, thus easily injuring normal cells by mistake. As a result, the demand for highly selective immunosuppressive drugs has gradually increased. Cyclosporine A has received much attention since its successful use in the treatment of organ anti-rejection, and researchers have again used cyclosporine A in animal studies and found it to be effective in human-like autoimmune myasthenia gravis in rats. Since then, cyclosporine A has been used clinically to treat human autoimmune diseases. However, mankind has never stopped for the use of immunosuppressive drugs to treat diseases. In 2019, a neo-coronavirus outbreak was reported in Wuhan, and studies on patients can reveal that granulocyte colony factor, interferon-inducible protein 10, monocyte chemotactic protein 1, macrophage inflammatory protein 1α, and TNF-α levels are higher in patients with severe COVID-19 than in those without severe disease, demonstrating that cytokine storms can have an impact on the extent of COVID-19 disease (Huang et al., 2020). The use of glucocorticoids for the treatment of refractory cytokine storms is now well documented and widely accepted, and researchers have put methylprednisolone into clinical use and found it to be effective in patients with COVID-19, with a mild discontinuation response. But then researchers conducted a retrospective study that included 309 patients with severe MERS and found that glucocorticoids, while suppressing the cytokine storm, also interfered with the immune response, resulting in reduced clearance of the pathogen (Arabi et al., 2018). Therefore, for patients with severe COVID-19, the principle of no glucocorticoid therapy for patients not meeting the indications for glucocorticoid application is currently adopted. Of course, seeing the dawn of immunosuppressive therapy for COVID-19, researchers will devote more time and effort to the development and use of immunosuppressive agents in the future.

Since the successful completion of the first kidney transplantation in the United States in the 1950s, immunosuppression has received more attention. In 1978, cyclosporine A was first used in clinical renal transplantation in the United Kingdom, and its combined application with hormonal drugs and azathioprine was called “triple therapy”, which greatly improved the 1-year survival rate of transplanted kidneys, which was a new milestone in the history of immunosuppressant development, from which immunosuppressive agents began to develop formally in the 1970s. By now, various types of immunosuppressive agents have been put into clinical application, and the immunosuppressive agents commonly used in clinical practice can be broadly classified into the following categories: 1). Glucocorticoids; 2). Cytotoxic drugs (e.g., cyclophosphamide); 3). Calmodulin inhibitors (e.g., cyclosporine, tacrolimus); 4). Macrolamines (e.g., macrolimus ethyl ester); 5). Chinese herbal immunosuppressive agents (e.g., total ginseng). Glucocorticoids have a wide range of pharmacological mechanisms of action and are the most commonly used immunosuppressive agents in clinical practice, and they can have inhibitory effects on multiple aspects of the immune response. Studies have shown that it inhibits the production of initial T lymphocytes and monocytes mainly by affecting the differentiation maturation, phenotype and function of dendritic cells in order to induce clonal incompetence or apoptosis of T lymphocytes, followed by the acquisition of immune tolerance to specific antigens. However, because of their excessive adverse effects, they are currently used mainly for the combination treatment of various diseases. In order to minimize the adverse effects caused by glucocorticosteroids during treatment, researchers are working to develop new synthetic immunosuppressive agents to reduce the amount of glucocorticosteroids. In the 1960s, azathioprine was successfully modified and first used successfully in organ transplantation (Nordham and Ninokawa, 2022), but after a long period of use it was found to have serious teratogenic and carcinogenic adverse effects. In addition to this, it is less selective for cells and still has a killing effect on cells that are proliferating faster. In order to find a solution to the strong organ toxicity of azathioprine, researchers have conducted studies on antimetabolic immunosuppressive agents and have succeeded in extracting mycophenolate esters from Penicillium spp. fungi, which inhibit the production of antibodies and control the rejection reactions that occur during organ transplantation. In 1995, mycophenolate was approved by the US FDA as an adjunct to cyclosporine A for the prevention of acute renal transplant rejection. There are still studies in China that can show that mycophenolate, as a highly selective immunosuppressant, can replace azathioprine in combination with hormones, and that the toxic effects in the treated organ are greatly reduced. Although mycophenolate esters have shown good improvements in their toxic effects, they still have a degree of significant gastrointestinal irritation (Omair et al., 2015), which limits their widespread use. Meanwhile, researchers succeeded in synthesizing cyclosporine in 1980 and then tacrolimus in 1984, and these mTOR inhibitors showed good immunosuppressive effects and greatly reduced adverse effects such as myelosuppression. In order to maximize the pharmacological effects of cyclosporine, researchers subsequently extracted and discovered sirolimus and imidazolibine, which were combined with cyclosporine, and found that the former and the latter had increased efficacy due to synergistic effects (Kahan, 1999). In the 21st century, more attention has been given to the development of monoclonal antibodies in order to further improve the targeting and duration of drug delivery. As a result, baximab and daximab were introduced. It was found that monoclonal drugs have a longer half-life, which can improve the dosing time to some extent.Because cyclosporine is widely used, but its nephrotoxic reactions are very obvious, so in recent years, researchers have tried to modify its structure, and finally obtained a new generation of calmodulin inhibitors - a cyclosporine derivative, vincristine, developed by Isotechnika, a Canadian company. This derivative has a better immunosuppressive effect and fewer adverse effects than cyclosporine and tacrolimus, and is widely used in autoimmune diseases such as psoriasis. In March 2021, it was also approved for the treatment of systemic lupus erythematosus in the US. In addition, studies are currently underway to investigate its use in COVID-19.

However, despite our previous adequate development of immunosuppressive drugs, there is still confusion and blind spots regarding the clinical use of immunosuppressive drugs. To date, the mechanism of efficacy of immunosuppressive agents has relied on their inhibition of lymphocyte proliferation and suppression of immune system-associated cytokine production. However, it is clear that there are many signaling pathways in the body that regulate cytokine production, thus complicating the impact of immunosuppressive agents at the molecular level. For example, as a common immune-related cytokine, its gene expression is regulated by many pathways, such as PP2A-GSK3β-MCL-1, PI3K-AKT-mTOR, MAPK and so on. On top of this, there are many targets on each signaling pathway, including proteins, kinases, DNA, etc. This complexity of organismal pathways greatly calls for a more refined classification of immunosuppressive agents, which can lead to greater clarity in drug use. To date, a number of immunosuppressive agents based on targets in signaling pathways have been introduced to the market. The first immunosuppressant to enter the public eye and clinical trials targeting the JAK-STAT pathway was tofacitinib, which is now approved by the FDA for autoimmune diseases such as rheumatoid arthritis. In addition to tofacitinib, rheumatoid arthritis has been pursued for targeted therapies for the past decades, and the advent of JAK inhibitors such as Filgotinib and Upadacitinib has led to promising treatment results in randomized controlled trials for this disease (Biggioggero et al., 2019). In the treatment of rheumatoid arthritis, sulforaphane can also exert its effects by inhibiting the MAPK pathway as well as the NF-κB pathway (see the MAPK pathway section in the main text for the specific mechanism of this part). Subsequently, studies on other autoimmune diseases on the JAK-STAT pathway and the development of targets have gradually increased, and a large amount of experimental data as well as clinical evidence support the possibility of developing immunosuppressive agents on this pathway, and clinical trials for some other indications, such as graft-versus-host rejection, transplantation, asthma, and lupus (Dowty et al., 2014; Okiyama et al., 2014; Furumoto et al., 2017), have been successfully conducted and obtained satisfactory results. While the JAK-STAT pathway has been methodically studied, many discoveries have been made in other pathways, for example, sirolimus, which acts in the PI3K-AKT-mTOR pathway, has long been approved as an immunosuppressant for the prevention of immune rejection of organ transplantation; berberine and curcumin, which act in the Keap1/ARE-Nrf2 pathway, have also demonstrated their anti-inflammatory efficacy, providing a basis for future The development of immunosuppressive agents has laid the foundation for future development. At present, there are already a variety of drugs that act on the target in the clinic, and the drugs that can act on the same target show similar physicochemical properties and conformational relationships, which to a certain extent has facilitated the development of new drugs and the transformation of old drugs. This new focus on further clarification of the pharmacological mechanisms as well as the targets of action of immunosuppressive drugs can be attributed in part to the prevalent disease complexity and the growing need for precision therapy and combination drug use. This article focuses on reviewing the latest research advances in immunosuppressive drugs, which will facilitate the clinical use of immunosuppressive drugs and improve the status of combinations. In addition, this review will introduce the common immune-related signaling pathways in the body, including JAK-STAT, NF-κB, PI3K-AKT-mTOR, MAPK, Keap1-Nrf2-ARE, and for each specific pathway, summarize the targets that immunosuppressive drugs can act on, and list the representative drugs that have been marketed in the clinic and in clinical trials.

### JAK-STAT Pathway

**FIGURE 1:**
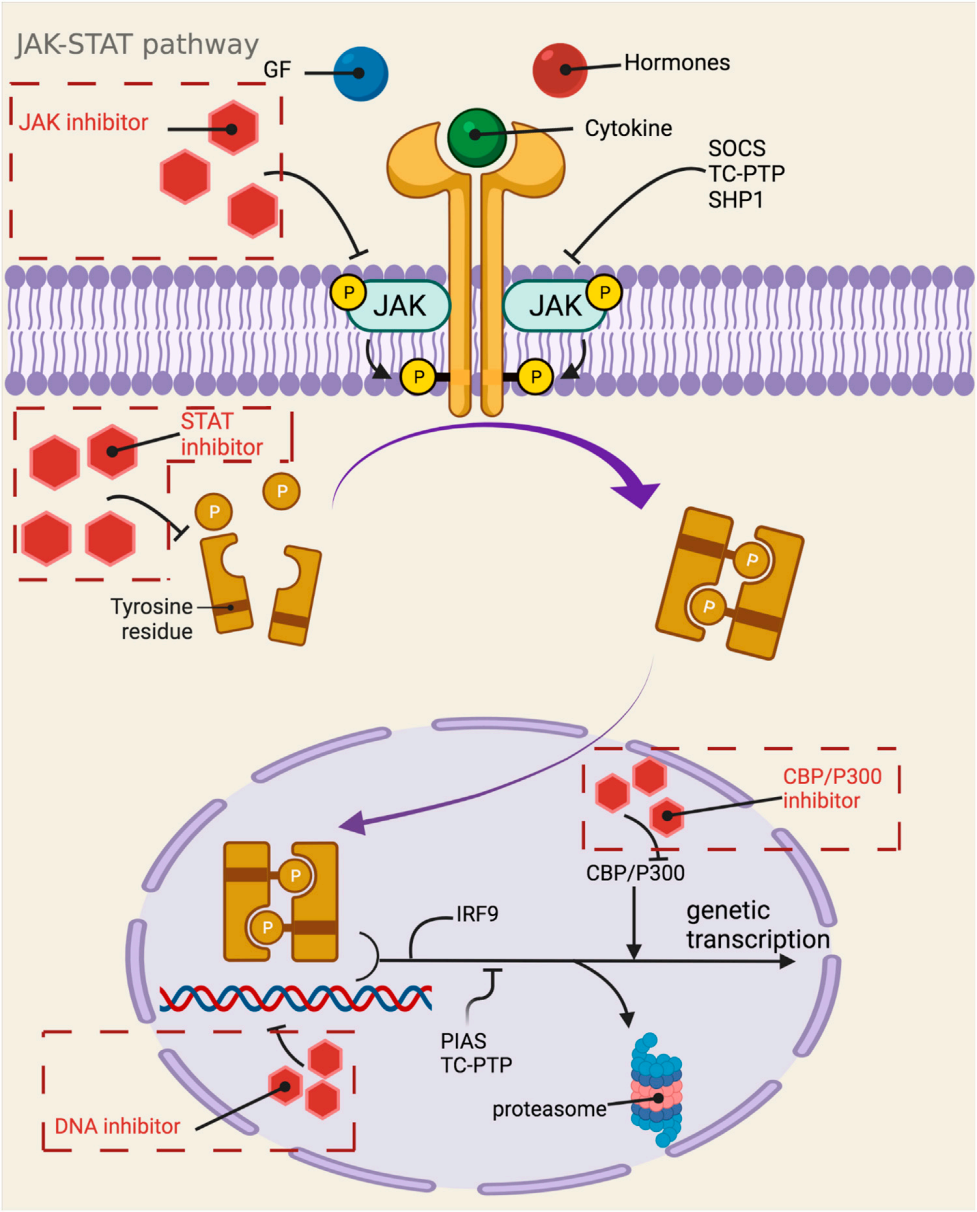
Mechanistic map of the JAK-STAT signaling pathway. CBP, calmodulin-binding peptide; GF, growth factor; IRF9, interferon regulatory factor 9; PIAS, protein inhibitor of activated STAT; SHP1, Src homology region 2 domain-containing phosphatase 1; SOCS, suppressor of cytokine siganaling; TC-PTP, T-cell protein tyrosine phosphatase. The figure is created with BioRender.com.

The JAK-STAT pathway was discovered by Darwell when he studied the signaling molecules required for the activation of target genes after the action of interferon (Darnell, 1998), and it is one of the main mechanisms regulating the production of cytokines. More than 50 cytokines, growth factors and hormones, such as interleukins, interferons, granulocytes/macrophages colony-stimulating factor, erythropoietin, and thrombopoietin, etc., by intercalating with transmembrane receptors, this brings them spatially close to JAK kinase, which changes the spatial conformation of JAK kinase and makes it susceptible to trans-activation. Activated JAK kinases promote STAT monomer phosphorylation and further dimerization, nuclear translocation, and binding to specific enhancer sequences of target genes in dimeric or more complex oligomeric forms, thus functioning as classical transcription factors. For example, STAT is involved in three types of transcription in immune cells, namely 1) general transcription, such as acetyltransferase, methyltransferase, p300, RNA polymerase, etc.; 2) transcription of some basic inflammation-related substances, such as IRF, NF-κB family transcription factors, etc.; 3) major transcription factors that are critical to follow the specification (Harrison, 2012; Villarino et al., 2015). The main three negative regulators involved in the negative regulation of JAK-STAT are: cytokine signaling inhibitory protein, activated STATs protein inhibitor, and protein tyrosine phosphatase. Among them, cytokine signaling inhibitory proteins negatively regulate JAK-STAT through three main mechanisms, including 1) binding to phosphorylated tyrosine on the receptor, which physically blocks the binding of STATs to the receptor; 2) binding to JAKs or the receptor, which blocks the activity of JAKs; 3) interaction of the SOCS box with the elonginB/C complex, which results in the degradation of JAKs and STATs, etc., are degraded via the ubiquitination pathway (Trengove and Ward, 2013). Activated STATs protein inhibitors achieve inhibition of STATs through two pathways: 1) binding to dimerized STATs and masking the DNA-binding region of STATs; and 2) binding to STATs monomers thereby hindering their dimerization.Protein tyrosine phosphatases block the activity of JAKs by dephosphorylating them through binding to JAKs and receptors, in addition to negatively regulating STATs (Quintás-Cardama and Verstovsek, 2013). CBP/p300 is a histone acetyltransferase that regulates the acetylation of STATs (Wieczorek et al., 2012), which would affect the signaling of NF-κB pathway, transcriptional activity and stability of STATs homodimers, and apoptosis (Ginter et al., 2013; Zhuang, 2013) (Figure 1). It was found that IL-6, IL-13, IL-22, granulocyte colony-stimulating factor and IFN exert their biological functions mainly by binding to JAK1, while IL-2, IL-4, IL-17, IL-15 and IL-21 exert their biological functions mainly by binding to JAK3 (O’shea et al., 2002). The pathways generally work together to regulate the cell through interactions such as mutual synergy, with large and small connections arising between each pathway. For example, because both STAT2 and PI3K proteins have SH2 structural domains on them, both can bind to these phosphorylated receptors and function when JAK proteins are activated and tyrosine residues on the receptors are phosphorylated. That is, the STAT2 protein on the JAK-STAT pathway and the PI3K protein on the PI3K-AKT-mTOR pathway have a synergistic effect, and they can jointly regulate signaling between cells (Ma et al., 2010). In addition, the JAK-STAT pathway can also interact with the MAPK/ERK pathway. A protein called Grb2, which plays an important role in the MAPK/ERK pathway, also has an SH2 structural domain and can also act on the phosphorylated receptor, thus acting synergistically with the two outer pathways (Xu and Qu, 2008). JAK-STAT can also indirectly activate the MAPK pathway through SOCS3, which can bind RasGAP, a negative regulator of the MAPK pathway, and thus exert a role in promoting the MAPK pathway (Herranz et al., 2012). Numerous studies have demonstrated that the activation of STATs is mostly accomplished not by JAKs but by receptor tyrosine kinases, by two mechanisms. One is that activation of some RTKs, including epidermal growth factor receptor and platelet-derived growth factor receptor, leads to the completion of STATs tyrosine phosphorylation via Src kinase. The other is that activation of the RTK/Ras pathway causes upregulation of mitogen-activated protein kinase activation, with MAPK specifically phosphorylating a serine (Ser) at the C-terminus of most STATs, and Ser phosphorylation greatly enhances the transcriptional activity of STATs (Coskun et al., 2013). Because of its involvement in the pathogenesis of many diseases, such as solid tumors, leukemia, lymphoma, and inflammatory diseases, a large number of studies on targeted therapies for this pathway have proliferated, with JAKs and STATs as the most common targets. For example, the STAT inhibitor Fludarabine has been approved for the treatment of B-cell chronic lymphocytic leukemia; the JAK inhibitor Upadacitinib has been approved for the treatment of moderately to severely active rheumatoid arthritis or active psoriatic arthritis; and the STAT inhibitor Stattic can exert an inhibitory effect on the auto-inflammatory response in myeloid, lymphatic and neuronal tissue compartments by inhibiting STAT3 (Alhazzani et al., 2021). In addition, this pathway can be inhibited by inhibiting the binding of STAT to DNA. For example, Rabies virus P protein can downregulate type I IFN production by inhibiting STAT1 binding to the DNA structural domain (Vidy et al., 2007); Phosphotyrosyl Peptides PY*LKTK,PY*L,AY*L (where Y* represents phosphotyrosine) can block this pathway by inhibiting STAT1 or STAT3 binding to DNA (You et al., 2020). CBP and p300 are essential transcriptional co-activators and histone acetyltransferases in cells, and overexpression or mutation of these two may cause the development of related diseases such as cancer, so inhibitors targeting them can also block the JAK-STAT pathway and thus play a therapeutic role. For example, Y08197 is a new inhibitor of this target with an indication of activity for the treatment of prostate cancer (Zou et al., 2019). Some of the JAK inhibitors and STAT inhibitors have been approved for marketing, while most of the drugs are still in the process of clinical trials or even animal studies, as shown in Table 1, which lists some of the drugs targeting the JAK-STAT pathway and their targets, indications, and stages of study.

**TABLE 1:** Targets in and inhibitors targeting the JAK-STAT pathway.

	Target	Agent	Phase	Indication	References
JAK inhibitor	JAK1	Filgotinib	approved	Rheumatoid Arthritis, Ulcerative Colitis	Dhillon and Keam (2020); Labetoulle et al. (2018)
		Upadacitinib	approved	Psoriatic Arthritis, Rheumatoid Arthritis, Atopic Dermatitis	Ferreira et al. (2020); Avci et al. (2021); Ross and Magrey, (2021)
		Abrocitinib	investigational	Atopic Dermatitis	Ferreira et al. (2020)
		Itacitinib	investigational	Graft Versus Host Disease	Schroeder et al. (2020)
		Solcitinib	investigational	Severe Ulcerative Colitis	De Vries et al. (2017)
	JAK2	Fedratinib	approved	Myeloid Proliferative Tumor, Myelofibrosis	Talpaz and Kiladjian, (2021)
		AZD1480	investigational	Solid Malignancies, Post-Polycythaemia Vera Myelofibrosis, Primary Myelofibrosis, Essential Thrombocythaemia Myelofibrosis	Hedvat et al. (2009)
		BMS-911543	investigational	Myeloproliferative Disorders	Purandare et al. (2012)
		Gandotinib	investigational	Myeloproliferative Neoplasms	Berdeja et al. (2018)
		Pacritinib	investigational	Acute Myeloid Leukemia	Komrokji et al. (2011)
		XL-019	investigational	Polycythaemia Vera, Myelofibrosis	Tam and Verstovsek, (2013)
		AG490	experimental	Subarachnoid Hemorrhage, Prostate Cancer	An et al. (2018)
	JAK3	Ritlecitinib	investigational	Rheumatoid Arthritis, Alopecia Areata	Montilla et al. (2019); Robinson et al. (2020)
	TYK2	Deucravacitinib	investigational	Psoriasis	Catlett et al. (2021)
		Ropsacitinib	investigational	Severe Plaque Psoriasis	Nogueira et al. (2020)
	JAK1/JAK2	Baricitinib	approved	Atopic Dermatitis	Wallace et al. (2018)
		Ruxolitinib	approved	Myeloid Proliferative Tumor, Graft Versus Host Disease	Chen et al. (2021)
		Momelotinib	investigational	Myelofibrosis, Post-polycythaemia Vera Myelofibrosis, Polycythaemia Vera, Post-essential Thrombocythaemia Myelofibrosis, Essential Thrombocythaemia, Acute Myeloid Leukemia	Tyner et al. (2010)
	JAK1/JAK3	Decernotinib	investigational	Rheumatoid Arthritis	Mahajan et al. (2015)
	JAK1/TYK2	Brepocitinib	investigational	Severe Ulcerative Colitis, Cicatricial Alopecia	Montilla et al. (2019); D'Amico et al. (2018)
	JAK2/TYK2	Nifuroxazide	experimental	Myeloid Proliferative Tumor	Nelson et al. (2008)
	JAK1/JAK2/JAK3	Oclacitinib	approved	Dermatitis (dogs)	Rynhoud et al. (2021)
		Tofacitinib	approved	Rheumatoid Arthritis, Psoriatic Arthritis, Juvenile Idiopathic Arthritis, Ulcerative Colitis	Kurasawa et al. (2018)
		Lestaurtinib	investigational	Acute Myeloid Leukemia	Knapper et al. (2017)
	JAK1/JAK2/JAK3/TYK2	Cerdulatinib	investigational	Lymphoid Leukemia, B-Cell Chronic Lymphocytic Leukemia	Coffey et al. (2019)
		Delgocitinib	investigational	Atopic Dermatitis	Dhillon, (2020)
		Gusacitinib	investigational	Atopic Dermatitis	Le et al. (2021)
		Peficitinib	investigational	Rheumatoid Arthritis	Qiu et al. (2019a)
STAT inhibitor	STAT1	Fludarabine	approved	Burkitt Lymphoma, Mantle Cell Lymphoma, Marginal Zone Non-Hodgkin Lymphoma	Izutsu et al. (2021)
	STAT3	Stattic	approved	Ankylosing Spondylitis	Schust et al. (2006)
		BP-1-102	investigational	Carcinogenesis, Neoplasm	Uchihara et al. (2019)
		FLLL32	investigational	Neoplasm	Onimoe et al. (2012)
		LLL-12	investigational	Inflammatory Disease, Acute Lung Injury	Lin et al. (2010a)
		Ochromycinone	investigational	Psoriasis	Boonlarppradab et al. (2013)
		OPB-31121	investigational	Leukemia Advanced cancer, Non-Hodgkin lymphoma, Multiple Myeloma, Hepatocellular Carcinoma, Solid Tumor	Leung et al. (2015a)
		OPB-51602	investigational	Nasopharyngeal Carcinoma, Advanced Cancer, Multiple Myelomas, Non-Hodgkin Lymphoma, Acute Myeloid Leukemia, Chronic Myeloid Leukemia, Malignant Solid Tumor	Leung et al. (2015a)
		Pyrimethamine	investigational	Chronic Lymphocytic Leukemia, Small Lymphocytic Leukemia, Malaria	Takakura et al. (2011)
		Resveratrol	investigational	Herpes Labialis Infections	Meng et al. (2021)
		STX-0119	investigational	Giloblastoma, Neoplasm	Siddiquee et al. (2007)
		Cryptotanshinone	experimental	Burkitt Lymphoma	Wu et al. (2020a)
		Cucurbitacin I	experimental	Neoplasm	Guo et al. (2018)
	STAT5	pimozide	approved	Motor and phonic tics	Nelson et al. (2011)
		Cpd1	investigational	Neoplasm, Polyploidy	Leung et al. (2015b)
		SF-1-088	investigational	Acute Myeloid Leukemia	Page et al. (2012)
	STAT3/STAT5	SH-4-54	experimental	Classic Hodgkin Lymphoma, Neoplasm	Cui et al. (2020)
	STAT5/STAT6	Panobinostat	approved	Multiple Myeloma	Eleutherakis-Papaiakovou et al. (2020)
	STAT3/TAX	Niclosamide	approved	Hymenolepiasis, Diphyllobothriasis	Kadri et al. (2018)
	STAT5/IL-2	CMD178	experimental	B-cell non-Hodgkin Lymphoma	Price-Troska et al. (2019)
	Apoptosis inducing Factor/STAT3	Atiprimod	investigational	Multiple Lymphoma	Coker-Gurkan et al. (2021)
CBP/P300 inhibitor	CBP/P300	Y08197	investigational	Castration-resistant Prostate Cancer	Zou et al. (2019)
		Acetylshikonin	investigational	Neoplasm, Inflammatory Disease	Hao et al. (2020)
		A-485	investigational	Inflammatory Disease, Osteoporosis	Huo et al. (2021)
		CBP30	investigational	Ankylosing Spondylitis, Psoriatic Arthritis	Hammitzsch et al. (2015)
		CCS1477	investigational	Haematological Malignancies	He et al. (2021)
		DC-CPin7	investigational	Leukemia	Chen et al. (2020a)
		DC-CPin711	investigational	Leukemia	Chen et al. (2020a)
		Ⅰ-CBP112	investigational	Leukemia	Picaud et al. (2015)
		EML425	experimental	Leukemia	Milite et al. (2015)
		Spermidine	experimental	Chronic Plaque-type Psoriasis, Rheumatoid Arthritis	Madeo et al. (2018)
		Zinc00542118	experimental	No Data	Zou et al. (2019)
		Zinc01428104	experimental	No Data	Zou et al. (2019)
		Zinc02635367	experimental	No Data	He et al. (2021)
		Zinc20617579	experimental	No Data	He et al. (2021)
		Zinc58215218	experimental	No Data	He et al. (2021)
		Zinc73744339	experimental	No Data	He et al. (2021)
DNA inhibitor	DNA binding	Angiotensin blockade	experimental	Inflammation, Proteinuric Kidney Disease	Chandar et al. (2007)
		Phosphotyrosyl Peptides	experimental	Leukemia, Inflammatory Disease	You et al. (2020)
		Rabies virus P protein	experimental	Inflammation, Leukemia, Cancer	Vidy et al. (2007)

Tofacitinib, developed by Pfizer, selectively inhibits JAK1 kinase, JAK2 kinase, and JAK3 kinase, and in a study of its stereochemical structure, Meyer et al. found that the chiral structure of tofacitinib determines its binding to the JAK receptor (Meyer et al., 2010). In their study, O’shea et al. found that the drug inhibited JAK1 kinase and JAK3 kinase to a greater extent than JAK2 kinase (O’Shea et al., 2015). In addition to this, the researchers found that tofacitinib had negligible activity against TYK2 (Zerbini and Lomonte, 2012). In 2020, the FDA approved it for the treatment of chronic idiopathic arthritis (including rheumatoid arthritis, psoriatic arthritis, ankylosing spondylitis, and juvenile idiopathic arthritis) through a risk assessment and mitigation strategy. In addition to rheumatoid arthritis and ulcerative colitis, tofacitinib can also be used to prevent immune reactions to organ transplants, as well as baldness and psoriasis, for which the application is still in clinical trials.After oral administration of tofacitinib, its absolute bioavailability is 74%, peak blood concentration is reached 1–2 h after dosing, and half-life is about 3 h. A high-fat diet does not affect AUC, but it decreases Cmax by 32%. When tofacitinib was administered intravenously, Vd = 87L and the drug was distributed to an equal extent in red blood cells and plasma. The metabolism of tofacitinib is mainly metabolized by the hepatic drug enzymes CYP3A4 and CYP2C19, and 30% is excreted by the kidneys in the form of the prototype drug. Kostovic et al. found that the pharmacological activity of the metabolites of tofacitinib was less than 10% of that of the prototype drug, proving that the pharmacologically active form of the drug is the prototype drug (Kostovic et al., 2017).

JAK inhibitors have been shown to increase the risk of herpes virus infection in the treatment of ulcerative colitis and psoriasis, demonstrating that JAK inhibitors have the ability to The side effect of reducing the immunity of the body and thus inducing infection. In addition, while tofacitinib has shown good efficacy, studies have statistically found that it also shows a risk of venous embolism. Although no thorough studies have shown that JAK inhibitors are harmful to pregnant or lactating women, long-term statistical observations have shown that they are teratogenic and should be avoided in pregnant and lactating women. In addition, the use of JAK inhibitors can also promote the course of hyperlipidemia, malignancy, and gastrointestinal perforation, thus suggesting the need for more caution in drug use (Agrawal et al., 2020). In addition to JAK inhibitors, STAT inhibitors can also exhibit side effects similar to those of JAK inhibitors, and Wong et al. found that two patients developed unusual infections with symptoms of herpes virus infection as well as acute epididymitis during a clinical trial of the novel STAT3 inhibitor OPB-51602, warning of the safety of its use (Wong et al., 2015).

### NF-κB Pathway

**FIGURE 2:**
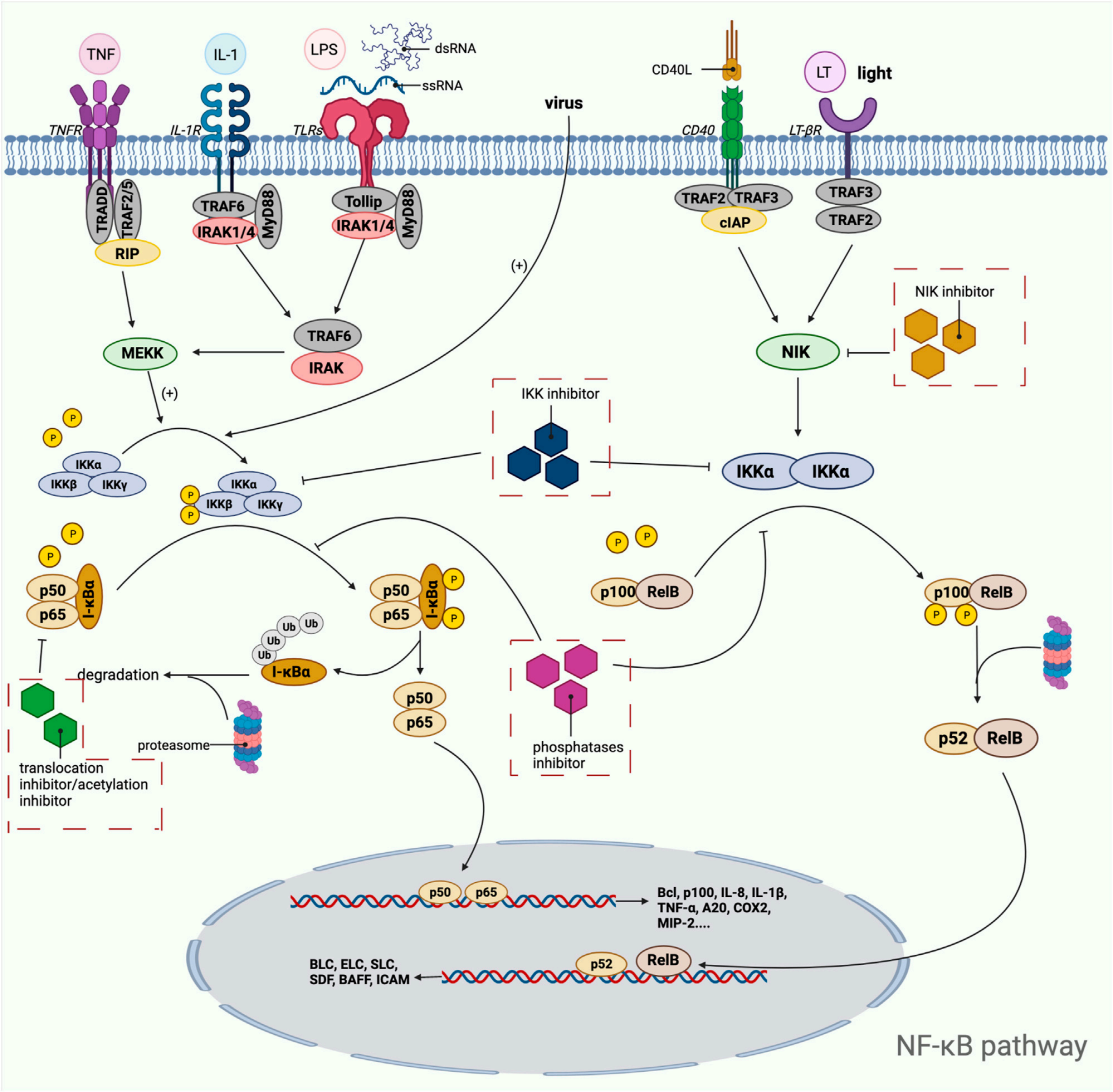
Mechanistic map of the NF-κB signaling pathway. BAFF, B-lymphocyte activating factor; Bcl, B-cell lymphoma; BLC, blood lactic acid; cIAP, cytoplasmic inhibitor apoptosisprotein; COX2, cyclooxygenase 2; ELC, ELongin C; ICAM, intercellular cell adhesion molecule; IL, interleukin; IRAK, interleukin-1 receptor-associated kinase; LPS, lipopolysaccharide; LT, Leukotriene; MIP, macrophage inflammatory protein; MyD: myeloid differentiation factor; RIP, receptor-interacting protein; SDF, stromal cell-derived factor; SLC, secondary lymphoid tissue chemokine; TNF, tumor necrosis factor; TRADD, TNF receptor-associated death domain; TRAF, tumor necrosis factor receptor associated factor The figure is created with BioRender.com.

NF-κB pathway is an important potential target pathway for drug treatment of diseases in human body. Since nuclear factor-κB is a common class of pro-inflammatory factors, this pathway is closely related to invasive response, inflammatory response, angiogenic and metastatic response. With the gradual research, it reflects the association between NF-κB pathway and many cancer and inflammatory diseases, such as viral infection, AIDS, arthritis, atherosclerosis, asthma, diarrhea, etc. (Gupta et al., 2010b). In mammals, NF-κB can be divided into five species, namely RelA (p65), RelB, c-Rel, NF-κB1 (p50) and NF-κB2 (p52). These five parts ensure their interconnection through the conserved Rel homology structural domains and even further form heterodimers or homodimers (Prescott and Cook, 2018). In the unactivated state, the NF-κB dimer binds tightly to the kappa B protein inhibitor in order to maintain the stability of the NF-κB dimer and inhibit its entry into the nucleus to interact with DNA. Activation of the NF-κB pathway can be divided into typical and atypical pathways, with the former involving IKK (a heterodimer composed of IKKα, IKKβ, IKKγ, and NEMO, of which IKKβ is the catalytic subunit), IκB, and NF-κB (e.g., p65/p50 heterodimer), including the TNF-α pathway, the IL-1β pathway, and the cellular stress pathway (Lin et al., 2010b). Among the classical pathways, the TNF-α pathway is the most well studied, and its activation pathway is as follows: after TNF-α and TNFR binding, IKK is aggregated in TNFR1 with the help of TRAF2/5 and receptor-interacting protein kinase, and then, receptor-interacting protein kinase mediates IKK phosphorylation to activate it, in which MEKK3 or TAK1 is also involved in the phosphorylation process (Devin et al., 2000). The catalytic subunit IKKβ is re-activated and then activates serine residues 32 and 36 of IκB, and further polyubiquitinates and degrades IκB by the proteasome. After this process, the NLS signal on p65 and p50 is exposed, promoting the p65/p50 nuclear translocation. Subsequently, p65/p50 binds to DNA and undergoes transcription, which is regulated by phosphorylation and acetylation of the p65 subunit, resulting in Bcl, p100, IL-8, IL-1β, TNF-α, A20, COX2, MIP-2, etc (Yang et al., 2001b) (Figure 2). The non-classical pathway includes CD40 pathway, LTβ pathway, etc. Unlike the classical pathway, the non-classical pathway mainly relies on NF-κB-inducible kinase (NIK) to activate IKKα, which in turn triggers the cleavage of p100 to p52. P52 in turn binds to RelB in a complex and undergoes nuclear translocation, binding to DNA and thus enhancing gene expression (Chen, 2005). In addition, through the cIAP protein, the classical and non-classical pathways can in turn be regulated by each other (Zarnegar et al., 2008). The current common targets and corresponding drugs are listed in Table 2.

**TABLE 2:** Targets in and inhibitors targeting the NF-κB pathway.

	Target	Agent	Phase	Indication	References
IKK inhibitor	IKKβ	Berberine	approved	Infection, Diarrheal	Pierpaoli et al. (2021); Yao et al. (2015); Pandey et al. (2008)
		Curcumin	approved	Pediatric Ulcerative Colitis, Neurodegenerative Disease, Vascular Dysfunction	Pricci et al. (2020); Lo Cascio et al. (2021)
		Nitric oxide	approved	Hypoxic Respiratory Failure	Reynaert et al. (2004); Manrique-Gil et al. (2021)
		Arsenite	investigational	Neoplasm	Kapahi et al. (2000); Ivanov and Hei, (2013)
		Withaferin A	investigational	Cognitive Dysfunction, Inflammation	Das et al. (2021); Logie and Vanden Berghe, (2020)
		ACHP	experimental	Multiple Myeloma, Adult T-cell Leukemia, HIV-1 Replication	Sanda et al. (2005); Sanda et al. (2006); Victoriano et al. (2006)
		AS602868	experimental	Acute Myelogenous Leukemia	Frelin et al. (2005)
		Bay 65–1942	experimental	KRAS-induced Lung Cancer, Chronic Pulmonary Inflammation, Ischemia–reperfusion Injury LPS-induced Neurotoxicity	Bassères et al. (2014); Ziegelbauer et al. (2005); Moss et al. (2007); Zhang et al. (2010)
		BI605906	experimental	Inflammation	Ba et al. (2019)
		Butein	experimental	Neoplasm	Tuli et al. (2021); Pandey et al. (2007)
		EqM	experimental	Leukemia, Colon, Kidney Cancer	Liang et al. (2006)
		IKK-16	experimental	Multiple Organ Failure Associated with Hemorrhagic Shock, Sepsis-associated Multiple Organ Dysfunction, Ventilation-induced Lung Injury, Acute Kidney Injury	Sordi et al. (2015); Coldewey et al. (2013); Shu et al. (2014); Johnson et al. (2017)
		IMD-0354	experimental	Chronic Lymphocytic Leukemia, Pancreatic Cancer, Adult T-cell Leukemia, Breast Cancer	Kanduri et al. (2011); Ochiai et al. (2008); Uota et al. (2012); Tanaka et al. (2006)
		LY2409881	experimental	Diffuse Large B-cell Lymphoma	Deng et al. (2015)
		MLN120B	experimental	Multiple myeloma, Arthritis	Hideshima et al. (2006); Schopf et al. (2006)
		PF 184	experimental	Arthritis	Sommers et al. (2009a); Sommers et al. (2009b)
		PHA-408	experimental	Arthritis, Chronic Obstructive Pulmonary Disease	Mbalaviele et al. (2009); Sommers et al. (2009c); Rajendrasozhan et al. (2010)
		pVHL	experimental	Neoplasm	Frew and Krek, (2008); Wang et al. (2016)
		SC-514	experimental	Rat Model of Inflflammation, Oral Squamous Cell Carcinoma, Osteoclast-related Disorders, Diabetic Neuropathy	Kishore et al. (2003); Johnson et al. (2014); Liu et al. (2013); Negi and Sharma, (2015)
		TPCA-1	experimental	Arthritis, Nasal Epithelium Inflammation, Glioma, Non-small Cell Lung Cancer, Chronic Obstructive Pulmonary Disease, Wet-type Age-Related Macular Degeneration	Podolin et al. (2005); Sachse et al. (2011); Du et al. (2012); Nan et al. (2014); Birrell et al. (2006); Lu et al. (2014)
	IKKα, IKKβ	Ainsliadimer A	experimental	Inflammation	Dong et al. (2015)
		BMS-345541	experimental	Arthritis, Colitis, Cardiac Graft Rejection, Acute T-lymphocytic Leukemia, Glioma, Prostate Cancer	Du et al. (2012); McIntyre et al. (2003); MacMaster et al. (2003); Townsend et al. (2004); Buontempo et al. (2012); Ping et al. (2016)
		BOT-64	experimental	Periodontal Diseases	Francis et al. (2020)
		CHS828	investigational	Solid Tumor	Olsen et al. (2004)
		Manumycin A	experimental	Neoplasm	Bernier et al. (2006); Macejová et al. (2020)
		Nimbolide	experimental	Neoplasm	Nagini et al. (2021); Gupta et al. (2010a)
		PS-1145	experimental	Multiple Myeloma, Diffuse Large B-cell Lymphoma, Graft-versus-host Disease, Tobacco Smoke-induced Pulmonary Inflammation	Hideshima et al. (2002); Lam et al. (2005); Vodanovic-Jankovic et al. (2006); Choi et al. (2016)
	IKK complex	5-fluorouracil	approved	Salivary Gland Cancer	Azuma et al. (2001)
		NBD peptide	experimental	Osteoclastogenesis, Inflammation	Jimi et al. (2004); May et al. (2000)
		vIL-10	experimental	Nasopharyngeal Carcinoma	Ren et al. (2016)
	IKKε	GSK 319347A	experimental	Reperfusion Injury	Zeng et al. (2019)
Multiple targets	IKKα, I-κBα	Betulinic acid	investigational	Cutaneous Metastatic Melanoma	Shankar et al. (2017); Gheorgheosu et al. (2014)
	IKKβ,I-κBα	Aspirin	approved	Pain, Fever, Inflammation	Alfonso et al. (2014); Kopp and Ghosh, (1994); Yin et al. (1998)
		Exisulind	investigational	Non-Small-Cell Lung Carcinoma, Prostate Cancer	Bunn et al. (2002); Webster and Leibovich, (2005)
		Sulindac sulphide	experimental	Neoplasm	Yamamoto et al. (1999); Ekanem et al. (2020)
	I-κBα, p65	Artemisinin	investigational	Schizophrenia, COVID-19	Uckun et al. (2021); Saeed et al. (2016); Wang et al. (2017)
	IKKβ, NF-κB	Arsenic trioxide	approved	Acute Promyelocytic Leukemia	Mathas et al. (2003); Yousefnia, (2021)
		Doxycycline	approved	Infections	Ogut et al. (2016)
	TANK-binding,IKKε	Amlexanox	approved	Non-Small-Cell Lung Cancer, Aphthous Ulcers	Reilly et al. (2013); Challa et al. (2016)
	p65, IKK,NIK	Mangiferin	experimental	Metastatic Melanoma	Takeda et al. (2016)
p65 acetylation inhibitor	p65	Gallic acid	approved	Diarrheal	Choi et al. (2009)
		Anacardic acid	experimental	Neoplasm	Sung et al. (2008); Hemshekhar et al. (2012)
protein phosphatases inhibitor		Cytosine arabinoside	approved	Acute Leukemia	Yang et al. (2001a); Sreenivasan et al. (2003)
		WIP1	experimental	No Data	Chew et al. (2009)
	I-κBα	Bortezomib	approved	Multiple Myeloma, Mantle Cell Lymphoma	Sunwoo et al. (2001); Lun et al. (2005); Khalesi et al. (2021)
		Phenylarsine oxide	experimental	Edema	Srivastava et al. (2016); Singh and Aggarwal, (1995)
	NF-κB	MG115	experimental	No Data	^N/A^
		MG132	experimental	Neoplasm	Guo and Peng, (2013)
		TLCK	experimental	Inflammation	Watanabe et al. (1986)
	NF-κB, RelA	TPCK	experimental	Inflammation	Watanabe et al. (1986)
N uclear translocation blockage		Dehydroxymethylepoxyquinomicin	experimental	Neoplasm	Lin et al. (2018); Umezawa, (2006)

Artemisinin is now publicly recognized as an effective drug for the treatment of malaria and has helped many countries around the world that are afflicted by the malaria disease. Mechanistically, artemisinin inhibits TNF-induced phosphorylation of NF-κB reporter factor I-κBα and its degradation by the proteasome, nuclear translocation of p65, and kinases upstream of IKK thereby achieving inhibition of the NF-κB pathway, thereby regulating genes related to cell proliferation, survival, invasion, and angiogenesis, such as Bcl, COX-2, MMP9, VEGF (Awasthee et al., 2019). As early as 2017, Wang et al. should have speculated on the possibility of artemisinin for the treatment of inflammation and cancer (Wang et al., 2017), and corresponding clinical trials are actively underway. In September 2021, clinical trials on the safety and efficacy of the herbal agent artemisinin for use in COVID-19 subjects were conducted, further demonstrating that its research on inflammation due to viral infections is still being explored.

The most common target in the NF-κB pathway is IKKβ, but IKKβ inhibitors are still less widely used in the clinic and are currently being developed at a lower rate, which has more to do with their safety profile. In fact, the NF-κB pathway is often considered as a “double-edged sword”, its anti-inflammatory response varies with the condition, for example, immune cells in tumors can play a dual role under the regulation of the pathway, which can be anti-inflammatory and anti-tumor response, but also can promote the development of tumor immune escape response (Ben-Neriah and Karin, 2011; Taniguchi and Karin, 2018). In addition, IKKβ inhibitors in the NF-κB pathway have more pronounced host differences, which may result from species variation or human host dependence (Prescott and Cook, 2018), which also makes it difficult to analyze the correlation between preclinical studies and clinical trials, and the difficulty of further drug development. More surprisingly, it was found that inhibition of IKKβ in certain cells or tissues exacerbates inflammation spontaneously, making drug use less safe and certain.Charles et al. found that IKKβ inhibited tumor growth in Colla2-expressing fibroblasts in a CAC model, but this was strictly dependent on increased secretion of HGF, and their speculation is that IKKβ/NF-κB may have different functions in different subpopulations of fibroblasts (Pallangyo et al., 2015). Therefore, it is important to have sufficient risk-taking ability when developing drugs for this pathway, as the good therapeutic effects of drugs in preclinical studies in animal models of disease may not be applicable to humans. Even if host differences are small, large individual differences may arise due to differences in the physiopathological conditions of the organism.

### PI3K-AKT-mTOR Pathway

**FIGURE 3:**
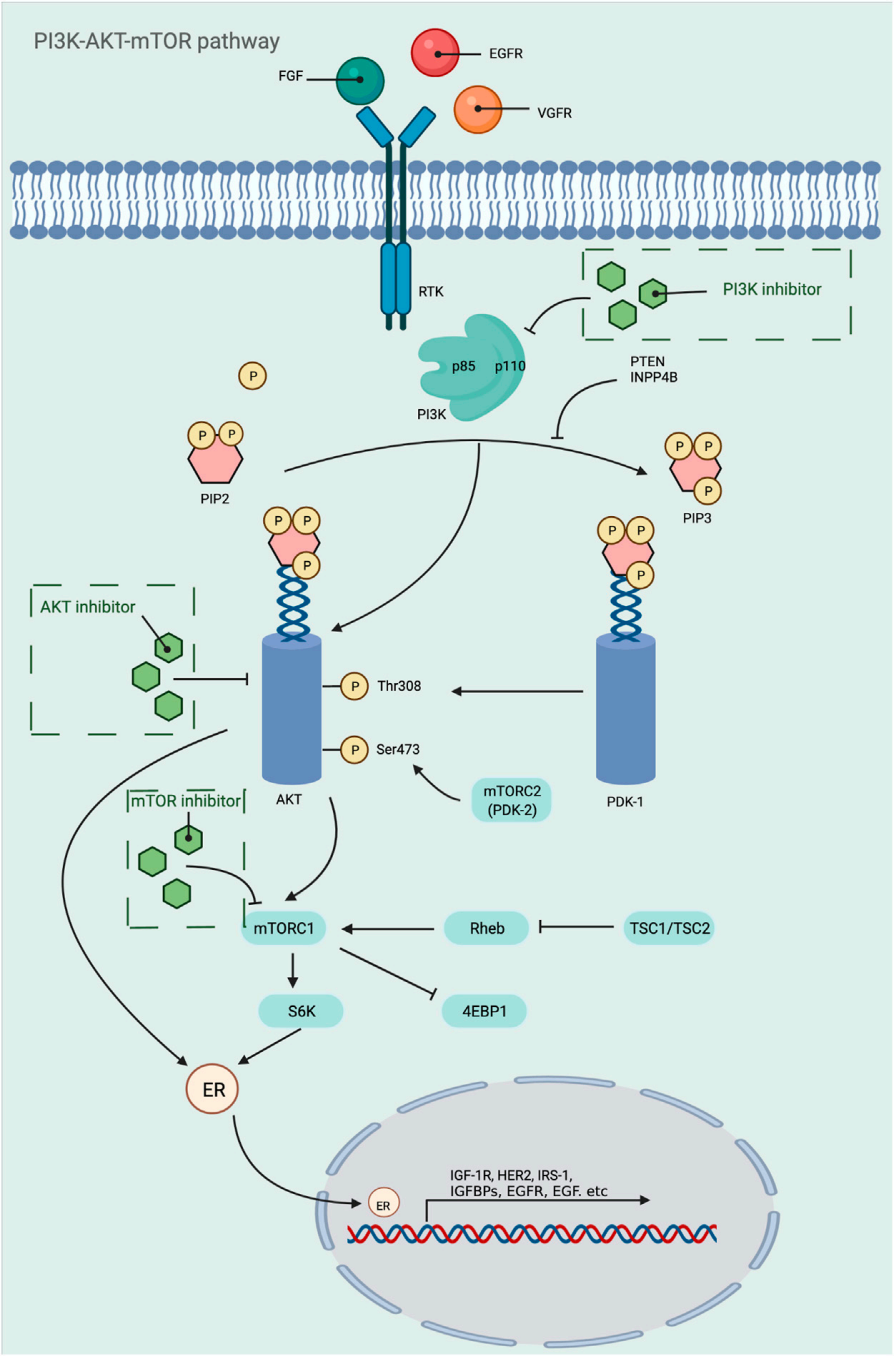
Mechanistic map of the PI3K-AKT-mTOR signaling pathway. EGF, epidermal growth factor; EGFR, epidermal growth factor receptor; FGF, fibroblast growth factor; HER2, human epidermalgrowth factor receptor-2; IGFBP, insulin-like growth factor-binding protein; IGF-1R, insulin-like growth factor 1 receptor; INPP4B, inositol polyphosphate 4-phosphatase typeⅡ; IRS-1, insulin receptor substrate 1; PDK, phosphoinositide-dependent protein kinase; PTEN, Phosphatase and tensin homologue deleted on chromosome 10; Rheb, Ras homolog enriched in brain; TSC, tumor stem cell; VGFR, vascular endothelial growth factor; 4EBP1, e IF4E-binding protein 1. The figure is created with BioRender.com.

PI3K/AKT/mTOR pathway is one of the most important signaling pathways in human body, which plays a crucial role in the activation of various downstream effector molecules and is involved in the regulation of cell proliferation, differentiation, apoptosis, autophagy, invasion and metastasis, etc. In response to stimulating factors such as growth factors and cytokines, residues on the transmembrane phosphorylated tyrosine kinase interact with the SH2 structural domain on PI3K, relieving the inhibitory effect of p58 on p110, i.e., the dimeric conformation is altered, leading to activation of PI3K (Osaki et al., 2004). In addition, PI3K activation can be accomplished by direct recognition and binding of Ras to p110. PI3K activation leads to the conversion of 3,4-bisphosphatidylinositol (PIP2) to 3,4,5-trisphosphatidylinositol (PIP3). The generated PIP3 recognizes each other with the PH structural domain of AKT, which results in the transfer of AKT from the cytoplasm to the cytosol, along with a conformational change of AKT, exposing threonine proteins as well as serine proteins. AKT is activated by the co-activation of Thr308 phosphorylation in the presence of PDK1 and Ser743 phosphorylation in the presence of PDK2 (Ma and Blenis, 2009). AKT is activated and translocated to the cytoplasm or nucleus, where it targets and regulates downstream signaling molecules, including mTOR. The nodular sclerosis complex-1 (TSC-1) and nodular sclerosis complex-2 (TSC-2) can form a dimeric complex that further inhibits the GTPase Rheb, which is required to stimulate mTOR activation, so the TSC-1/TSC-2 complex has an inhibitory effect on mTOR activation. However, the activation of AKT can release the inhibition of mTOR by TSC-1/TSC-2, thus allowing the smooth activation of mTOR. In addition, AKT can also act directly on mTOR1 to activate mTOR (Huang and Manning, 2008). Phosphorylated mTOR further regulates ribosomal S6 protein kinase (S6K) and the eukaryotic initiation factor 4E-binding protein 1 (4E-BP1), which promotes phosphorylation of ribosomal S6 proteins and inactivates 4E-BP1 by phosphorylation. This leads to the synthesis of ribosomal proteins and the initiation of protein translation processes, respectively (Wan and Helman, 2007) (Figure 3). Since this pathway is shown to be dysregulated in various tumors and inflammatory diseases, this shows great promise for the study of this pathway. Researchers have identified and developed a number of drugs that target this pathway, which can be broadly classified as PI3K inhibitors, AKT inhibitors, mTOR inhibitors, and dual PI3K/mTOR inhibitors. These inhibitors inhibit the activation of PI3K, AKT, and mTOR by inhibiting these three targets, which in turn is closely linked to the degree of tumor metastasis and related disease progression. A large number of preclinical studies and clinical trials on these inhibitors exist today to ensure safety and efficacy during drug use. Table 3 provides a summary of some of the inhibitors for different targets, with information about their indications.

**TABLE 3:** Targets in and inhibitors targeting the PI3K-AKT-mTOR pathway.

	Target	Agent	Phase	Indication	References
PI3K inhibitor	PI3Kα	Alpelisib	approved	Advanced or Metastatic Breast Cancer	Chang et al. (2021)
		GDC-0077	investigational	Breast Cancer	Song et al. (2021)
		Serabelisib	investigational	Metastatic Clear Cell Renal Cell Carcinoma, Breast Cancer, Neoplasm	Patel et al. (2019)
	PI3Kβ	AZD-8186	investigational	Breast and Prostate Tumors	Hancox et al. (2015)
		GSK2636771	investigational	Cancer, Lymphoma, Solid Neoplasm, Recurrent Solid Neoplasm, Advanced Malignant Neoplasm	Mateo et al. (2017); Sarker et al. (2021)
	PI3Kγ	Eganelisib	investigational	Locally Advanced HPV+ and HPV- Head and Neck Squamous Cell Carcinoma	Qiu et al. (2019b)
	PI3Kδ	Idelalisib	approved	Chronic Lymphocytic Leukemia, Relapsed Follicular B-cell non-Hodgkin Lymphoma, Relapsed Small Lymphocytic Lymphoma	Zirlik and Veelken, (2018)
	pan-PI3K	Copanlisib	approved	Relapsed Follicular Lymphoma	Munoz et al. (2021)
		Buparlisib	Investigational	Lymphoma, Metastases, Lung Cancer, Solid Tumors, Breast Cancer	Geuna et al. (2015); Xing et al. (2021)
		CH-5132799	investigational	Solid Tumors	Ceccarelli et al. (2021)
		Pictilisib	investigational	Solid Cancers, Breast Cancer, Advanced Solid Tumors, Metastatic Breast Cancer, non-Hodgkin Lymphoma	Shapiro et al. (2020); Li et al. (2020)
		Sonolisib	investigational	Glioblastoma, Prostate Cancer, Advanced Solid Tumors, Advanced BRAF-mutant Cancers, Non-Small Cell Lung Cancer	Harder et al. (2019); Levy et al. (2014)
		ZSTK474	investigational	Neoplasm	Muthiah and Callaghan, (2017)
AKT inhibitor	AKT	Afuresertib	investigational	Cancer, Neoplasms, Haematologic	Yamaji et al. (2017)
		Erufosine	experimental	Neoplasms	Tzoneva et al. (2020)
		MK2206	investigational	Relapsed or Refractory Diffuse Large B cell Lymphoma Non-Small Cell Lung Cancer	Hirai et al. (2010)
		Perifosine	investigational	Solid Tumors, Multiple Myeloma, Leukemia (unspecified), Lung Cancer, Brain Cancer	Block et al. (2010)
		SR13668	investigational	Neoplasms	Banerjee et al. (2013)
		Uprosertib	investigational	Breast Neoplasm	Jabbarzadeh Kaboli et al. (2020)
	PKA	A-443654	experimental	Leukemia, Neoplasms	de Frias et al. (2009); Han et al. (2007)
		A-674563	experimental	Carcinogenesis, Neoplasms	Chorner and Moorehead, (2018)
	AKT/PKB/p70 S6K	AT7867	investigational	Pancreatic Diseases, Thymoma, Neoplasms	Kimura et al. (2020); Grimshaw et al. (2010)
	AKT/PKB/p70 S6K/ROCK	AT13148	investigational	Hypotension, Neoplasms	Pal et al. (2020)
	ATP binding pocket	Capivasertib	investigational	Metastatic Breast Cancer	Zhu et al. (2021a)
		GSK690693	investigational	Tumor, Cancer, Lymphoma	Levy et al. (2009)
		Ipatasertib	investigational	Cancer, Neoplasms, Solid Cancers, Breast Cancer, Gastric Cancer	Shapiro et al. (2021)
		CCT128930	experimental	Osteosarcoma, Neoplasm	Sun et al. (2020)
		H-8	experimental	No Data	Nitulescu et al. (2016)
		H-89	experimental	No Data	Nitulescu et al. (2016)
		NL-71–101	experimental	No Data	Nitulescu et al. (2016)
mTOR inhibitor	mTORC1/mTORC2	AZD8055	investigational	Cancer, Lymphomas, Solid Tumors, Malignant Glioma, Brainstem Glioma	Chresta et al. (2010)
		Ku-0063794	investigational	Neoplasm	García-Martínez et al. (2009)
		OSI-027	investigational	Solid Tumor, Lymphoma	Bhagwat et al. (2011)
		PP242	investigational	Neoplasms, Leukemia	Feldman et al. (2009)
		Vistusertib	investigational	Neoplasm	Huo et al. (2014); Pancholi et al. (2019)
		PP30	experimental	Pneumoperitoneum, Leydig Cell Tumor	Feldman et al. (2009)
		Torin1	experimental	Tuberous Sclerosis, Neoplasm	Thoreen et al. (2009)
	mTORC1	Everolimus	approved	Breast Cancer	Ballou and Lin, (2008)
		Sirolimus	approved	Organ Transplantation	Ballou and Lin, (2008)
		Temsirolimus	approved	Renal Cell Carcinoma	Ballou and Lin, (2008)
		Ridaforolimus	investigational	Solid Tumors, Sarcoma, Cancer/Tumors (unspecified), Endometrial Cancer, Prostate Cancer, Bone Metastases	Spreafico and Mackay, (2013)
		Olcorolimus	experimental	Asthma	Eynott et al. (2003)
		Zotarolimus	experimental	Thrombosis, Myocardial Infarction	Ballou and Lin, (2008)
	ATP binding	WAY-600	experimental	Neoplasm	Yu et al. (2009)
		WYE-354	experimental	Neoplasm	Yu et al. (2009)
		WYE-687	experimental	Neoplasm, Severe Combined Immunodeficiency	Yu et al. (2009)
PI3K/mTOR dual inhibitor	PI3K/mTOR	Apitolisib	investigational	Solid Cancers, Breast Cancer, Prostate Cancer, Renal Cell Carcinoma, Endometrial Carcinoma	Dolly et al. (2016)
		Bimiralisib	investigational	Breast Cancer	Yang et al. (2020)
		Dactolisib	investigational	Cancer, Solid Tumor, Renal Cancer, Breast Cancer, Cowden Syndrome	Maira et al. (2008)
		XL765	investigational	Breast Cancer, Solid Tumor, Malignant Glioma	Molckovsky and Siu, (2008)
		GNE477	experimental	Cancer	Heffron et al. (2010)

The PI3K/AKT/mTOR pathway is associated with many diseases, and there are many factors that affect the physiological status of the human body in addition to the three critical targets of PI3K, AKT, and mTOR. For example, PTEN, a tumor suppressor, blocks the activation of PI3K/AKT/mTOR pathway by inhibiting the transition from PIP2 to PIP3. It was found that PTEN knockout mice had abnormally high levels of PIP3 compared to normal mice, and AKT remained continuously activated, which in turn induced tumorigenesis (Papa et al., 2014; Haddadi et al., 2018). In addition, the knockout mice also showed hypoglycemia, suggesting that it also has some effect on blood glucose regulation *in vivo* (Nguyen et al., 2006).

Sirolimus is a first-generation mTOR inhibitor targeting mTORC1, which specifically acts on mTORC1, causing phosphorylation of the carboxy terminus of mTOR and loss of catabolic activity, blocking the immune response triggered by interleukin-2, interleukin-15 and CD28/B7 co-stimulatory pathway to activate mTOR. It can inhibit the growth and proliferation of immune cells by keeping them in the G1/S phase, in addition to inhibiting the synthesis of immune molecules such as interleukin-1. Nowadays, it is mainly used in clinical practice to prevent rejection after organ transplantation and to treat autoimmune diseases (Weichhart, 2018; Li et al., 2019). Nowadays, indications for sirolimus are gradually being developed. For example, in primary immune thrombocytopenia, decreased Treg cell levels are the main cause of refractory/recurrent ITP, which provides a theoretical basis for sirolimus treatment of ITP. After a randomized group trial by Li (Cuker and Neunert, 2016) et al. showed that sirolimus significantly improved remission rates as well as platelet counts in patients with ITP (Li et al., 2013). Jasinski et al. included 12 patients with ITP and switched to sirolimus combined with hormone therapy after conventional treatment failed and found that the patients’ cure rate was greatly improved and no significant adverse effects were observed during the use of the drug (Jasinski et al., 2017), further establishing the possibility of sirolimus for the treatment of immune thrombocytopenia. Blood levels peaked after 1 h in healthy subjects after administration of sirolimus. In stable renal transplant patients, the half-life of sirolimus can be monitored to be approximately 46–78 h. The study demonstrated that the mean bioavailability of sirolimus tablets was 27% higher compared to the solution and that the plasma protein binding of sirolimus was 92%, with approximately 97% being bound to serum proteins. The metabolism of sirolimus is mainly carried out by CYP3A4 enzyme, and after hepatic metabolism, it is excreted out of the body mainly in the feces, and only a small amount is excreted out of the urine. Regarding the safety of sirolimus, the most common side effect was found to be grade 1–2 mucositis, in addition to many other adverse reactions such as stomatitis, oral ulcers, hyperlipidemia, infection and hepatic impairment, but it was found that most of these adverse reactions are dose dependent and the symptoms of these adverse reactions can be alleviated when the blood concentration of sirolimus decreases (Bride et al., 2016; Long et al., 2018; Chen et al., 2020b; Feng et al., 2020). In addition to mTOR inhibitors, other targets on the PI3K-AKT-mTOR pathway still exist, such as PI3K. Alpelisib was the first PI3Kα inhibitor identified and is currently approved and widely used for the treatment of breast cancer, in addition to being approved by the FDA in 2020 as a fast track for the treatment of the PIK3CA-associated overgrowth disease spectrum. During preclinical modeling, it was found to inhibit two common mutation sites of PI3K (H1047R and E545K) (Fritsch et al., 2014), in addition to having a dual mechanism of action, i.e., simultaneous inhibition of PI3K as well as induction of p110α degradation (Chang et al., 2021), and these highlight the possibility of its development as an immunosuppressive agent.

### Keap1-Nrf2-ARE Pathway

**FIGURE 4:**
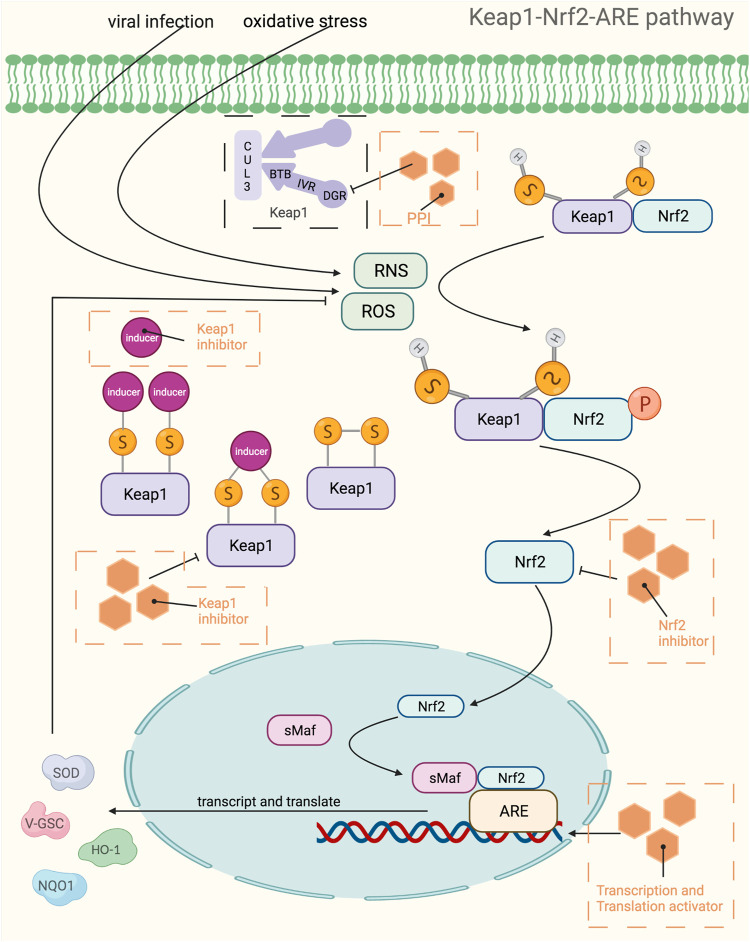
Mechanistic map of the Keap1-Nrf2-ARE signaling pathway. BTB, broad complex, tramtrack and bric-à-Brac; CUL3, cullin 3; DGR, double glycine repeat; HO-1, Heme Oxygenase-1; IVR, intervening region; NQO1, NAD(P)H:quinone oxidoreductase 1; PPI, proton pump inhibitors; RNS, reactive nitrogen species; ROS, reactive oxygen species; sMaf, specific macrophage activation factor; SOD, superoxide dismutase; GSC, glioblastoma stem cell. The figure is created with BioRender.com.

The Keap-Nrf2-ARE pathway is a key pathway for cellular resistance to oxidative stress and has neutralizing, antioxidant as well as detoxifying effects because it regulates antioxidant enzymes and phase II detoxifying enzymes. This pathway is often used as a drug target to treat a variety of diseases, including neurodegenerative diseases, cancer, cardiovascular diseases, respiratory diseases, and various inflammatory conditions. For example, quercetin can enhance brain function in learning memory by upregulating the expression of Nrf2 and the antioxidant gene OH-1 thereby reducing oxidative stress in the brain (Silva et al., 2017), and resveratrol can effectively protect against oxidative damage due to renal hyperglycemia mediated by upregulating antioxidant genes including catalase (CAT), GSH-Px, SOD and HO-1 (Muselin and Cristina, 2019). The Keap1-Nrf2-ARE pathway consists of three core components, Keap1, Nrf2, and ARE, which are activated to transcribe and express downstream antioxidant genes through various targets in this pathway. Under normal physiological conditions, most of Nrf2 couples to the Neh2 structural domain on Keap1 in an overall stable intracellular environment and anchors to the cytoplasm in conjunction with cytoplasmic agonist proteins. In contrast, under oxidative stress as well as stimulation by electrophile substances, the electrically sensitive cysteine structure on Keap1 protein is mutated, resulting in a conformational change of Keap1 (Wakabayashi et al., 2004). Changes in the structure of Keap1 cause dissociation between it and Nrf2, and the activated Nrf2 translocates into the nucleus and binds to Maf proteins in the nucleus to form a heterodimer, which in turn binds to the ARE and regulates transcription of downstream target genes (Magesh et al., 2012). Besides, the variation of Keap1 structure can also reduce the degradation of Nrf2 ubiquitination, i.e., it can make the Nrf2 protein more stable (Kensler et al., 2007). The increased stability of Nrf2 protein is also associated with the phosphorylation of its degron region under oxidative stress and the resulting conformational change, which prevents its recognition by E3 ubiquitinylation ligase, thereby weakening the recognition of Nrf2 by the protease and activating the intrinsic transcriptional activity of Nrf2.It has been shown that Nrf2, upon dissociation from Keap1, also synergistically promotes activation of the intrinsic transcriptional activity of Nrf2 by its two active regions, Neh4 and Neh5 coactivators, CBP proteins (Nguyen et al., 2005). The downstream target proteins regulated by Nrf2 have now been shown to fall into several categories: phase II metabolic enzymes, antioxidant proteins/enzymes, proteasomal/molecular partners, anti-inflammatory factors, and phase III metabolic enzymes (i.e., drug transporters). Among them, the main ones of wide interest are quinone oxidoreductase-1 (NQO1), heme oxygenase-1 (HO-1) and γ-glutamylcysteine synthetase (γ-GCS), which exert antioxidant effects and thus treat related diseases (Figure 4). Nrf2 and NF-κB pathway are key factors sensitive to redox homeostasis, and the interaction mechanism between the two may lead to a variety of diseases, such as pharmacogenic liver diseases. When a drug stimulates oxidative stress in cells, Nrf2 increases the expression of antioxidant enzymes and GSH, neutralizes ROS in hepatocytes, helps to reduce the degree of oxidative stress, and inhibits NF-κB expression; on the contrary, if Nrf2 expression is absent, NF-κB is more active at this time, leading to the accumulation of inflammatory factors (Ganesh Yerra et al., 2013). Therefore, the use of Nrf2 activators and Nrf2 inhibitors affects not only the operation of the Keap1/ARE-Nrf2 pathway, but also the NF-κB pathway. Currently, a large number of drugs targeting this pathway have entered clinical trials, while some of them only have pharmacological indications, Table 4 provides a summary of some of the drugs targeting the Keap1-Nrf2-ARE pathway, with a brief overview of their indications and stages of study, etc.

**TABLE 4:** Targets in and inhibitor targeting the Keap1-Nrf2-ARE pathway.

	Target	Agent	Phase	Indication	Reference
Nrf2 inhibitor	Nrf2	All-trans-retinoic acid	approved	Acute Promyelocytic Leukemia, Neoplasm	Giuli et al. (2020); Wang et al. (2007)
		Ascorbic acid	approved	Atrial Fibrillation, Stroke	Chen et al. (2020c); Tarumoto et al. (2004)
		Bexarotene	approved	Cutaneous T-Cell Lymphoma, Mycosis Fungoides	Pileri et al. (2013); Wang et al. (2013)
		Clobetasol propionate	approved	Psoriasis	Del Rosso, (2020); Choi et al. (2017)
		Dexamethasone	approved	Multiple Myeloma	Eleutherakis-Papaiakovou et al. (2019); Ki et al. (2005)
		Halofuginone	investigational	Fibrosis	Jain et al. (2021); Tsuchida et al. (2017)
		Luteolin	investigational	Neoplasm	Franza et al. (2021); Tang et al. (2011)
		AEM1	experimental	Neoplasm	Hoffmann et al. (2014); Bollong et al. (2015)
		Brusatol	experimental	Neoplasm	Xie et al. (2021); Ren et al. (2011)
		Malabaricone-A	experimental	Leukemia	Manna et al. (2015)
		ML385	experimental	Inflammation	Singh et al. (2016)
		Ochratoxin A	experimental	Kidney Diseases (induce)	Limonciel and Jennings, (2014)
		Trigonelline	experimental	Diabetes Mellitus	Arlt et al. (2013)
		Wogonin	experimental	Neoplasm	Huynh et al. (2020); Zhong et al. (2013)
Transcription and Translation activator	DNA	Lycopene	approved	Adrenal Cortex Diseases, Prostrate Neoplasm, Atherosclerosis	Mirahmadi et al. (2020); Costa-Rodrigues et al. (2018)
		Andrographolide	investigational	Ulcerative Colitis	Paul et al. (2021)
		Rosmarinic acid	investigational	Cerebral Hemorrhage, Extrahepatic Cholestasis, Encephalomalacia, Nervous System Disorders	Ghasemzadeh and Hosseinzadeh, (2020)
		L-F001	experimental	CNS Inflammation	(Chen et al., 12017)
		3H-1,2-Dithiole-3-thione	experimental	Neoplasm	Park et al. (2008)
Keap1 modification	Keap1	Beta carotene	approved	Reduction of Photosensitivity in patients with Erythropoietic Protoporphyria and other Photosensitivity Diseases, Macular Degeneration	Johra et al. (2020)
		Curcumin	approved	Pediatric Ulcerative Colitis, Neurodegenerative Disease, Vascular Dysfunction	Pricci et al. (2020); Lo Cascio et al. (2021)
		Plumbagin	investigational	Metastatic Castration-Resistant Prostate Cancer	Yin et al. (2020)
		Tert-butylhydroquinone	investigational	Hepatocellular Carcinoma, Acne Vulgaris	Li et al. (2014)
		Allyl sulfide	experimental	Hepatitis	Li et al. (2018)
PPI		Graveoline	experimental	Phototoxic Dermatitis	Sampaio et al. (2018)
		Tetrahydroisoquinoline	experimental	Parkinson Disease	Abe et al. (2005); Richardson et al. (2015); Jnoff et al. (2014)
		Thiopyrimidine	experimental	Neoplasm	Syam et al. (2019); Marcotte et al. (2013)
Nrf2 activator		Caffeine	approved	Neoplasm	Bors et al. (2018)
		Cinnamaldehyde	approved	Neoplasm	Aminzadeh et al. (2021)
		Dimethyl fumarate	approved	Relapsing Remitting Multiple Sclerosis	Valencia-Sanchez and Carter, (2020)
		Ethyl ferulate	approved	Alzheimer Disease, Inflammation	Wu et al. (2021);Mohmmad Abdul and Butterfield, (2005)
		Ferric pyrophosphate citrate	approved	Iron Deficiency, Enemia	Mazgaj et al. (2020)
		Bardoxolone	investigational	Neoplasm	Ju et al. (2021)
		Bardoxolone methyl	investigational	Neoplasm	Chien et al. (2021)
		Epigallocatechin gallate	investigational	Neoplasm	Tauber et al. (2020)
		Genistein	investigational	Prostate Neoplasm, Breast Neoplasm	Jaiswal et al. (2019); Bhat et al. (2021)
		Paeonol	investigational	Atherosclerosis, Hepatocellular Carcinoma	Vellasamy et al. (2021); Chen et al. (2012)
		Phenethyl Isothiocyanate	investigational	Leukemia, Lung Cancer, Lymphoproliferative Disorders	Wang et al. (2018); Sun et al. (2019); Gupta et al. (2014)
		Piperine	investigational	Neoplasm, COVID-19	Quijia and Chorilli, (2021); Miryan et al. (2021)
		Quercetin	investigational	Neoplasm	Zhao et al. (2021); Zang et al. (2021)
		Xanthohumol	investigational	Breast Neoplasm, Hepatocellular-Carcinoma, Colorectal Neoplasms, Lymphatic Metastasis	Gieroba et al. (2020); Seitz et al. (2021); Krajka-Kuniak et al. (2013)
		Baicalin	experimental	Brain Ischemia, Hepatitis B	Liang et al. (2017); Yang et al. (2021)
		Caffeic acid phenethyl ester	experimental	Neoplasm	Lv et al. (2021)
		Carnosic acid	experimental	Mitochondrial Diseases	de Oliveira, (2018)
		Chalcone	experimental	Breast Neoplasm, Lung Neoplasm	Komoto et al. (2021); Dong et al. (2018)
		Coumarin	experimental	Hemorrhage, Chemical and Drug Induced Liver Injury	Verhoef et al. (2010); Zhang et al. (2020)
		Kaempferol	experimental	Colorectal Cancer, Cardiovascular Diseases	Imran et al. (2019); Wang et al. (2019)
		Procyanidin	experimental	Atherosclerosis, Periodontitis	Govindaraj et al. (2011)
		Piperlongumine	experimental	Schistosomiasis, Neoplasm	Mengarda et al. (2020); Zhu et al. (2021b)
		Taxifolin	experimental	Hemolysis, Hepatocellular Carcinoma	Hapner et al. (2010); Butt et al. (2021)
		Vanillic acid	experimental	Neoplasm, Inflammation	Brimson et al. (2019)

All-trans retinoic acid is an agonist of retinoic acid receptor alpha, which is a nuclear receptor agonist that inhibits the transcriptional activity of Nrf2 (Natalia et al., 2019). It was shown that all-trans retinoic acid could interfere with the dimerization between bZIP factors and small Maf proteins, which would severely affect the binding between Nrf2 and DNA (Nioi et al., 2003). In addition to this, all-trans retinoic acid can also bind to RARs as a ligand for RARs, further leading to the subnuclear relocalization of Nrf2 and affecting the delocalization of transcriptional intermediate factor 1 to the centromeric heterochromatin region (Cammas et al., 2002), all of which would demonstrate the inhibitory effect of all-trans retinoic acid on Nrf2. The treatment of acute promyelocytic leukemia is its most common use today and has been approved by the FDA for clinical treatment as early as a decade ago. All-trans retinoic acid exerts its therapeutic effect by inducing terminal dichotomization in leukemic cell lines as well as APL cells, and the first study in France found that patients with acute promyelocytic leukemia using all-trans retinoic acid had few reactions such as primary resistance and alopecia (Degos and Wang, 2001). In 2021, the FDA approved all-trans retinoic acid in combination with benzoyl peroxide in cream form for the treatment of acne vulgaris in patients 9 years of age and older, demonstrating its new use and enabling a wider range of applications. In addition to the topical treatment of acne vulgaris, it can also be used to treat psoriasis, congenital ichthyosis, ichthyosis vulgaris, lamellar ichthyosis, phyllodes keratoses and other skin conditions as well as to improve fine lines, hyperpigmentation, roughness and symptoms associated with photodamage. However, to ensure the safety and efficacy of the treatment, the related diseases are still in the clinical trial or recruitment stage. When treating skin diseases, only 1%–31% of the drug is absorbed by the skin, and when combined with benzoyl peroxide, the degree of absorption is again influenced by age. All-trans retinoic acid has a half-life of 0.5–2 h and is metabolized in the body mainly by the liver, with the end product being retinoic β-glucuronide. In safety studies of all-trans retinoic acid, it has been found that some patients develop leukocyte activation syndrome (Castaigne et al., 1990) and drug resistance (Frankel et al., 1992) after treatment of acute promyelocytic leukemia, and to minimize the occurrence of side effects, all-trans retinoic acid is now commonly used in combination with intensive chemotherapy or by high-dose injections of glucocorticoids to resist side effects (Degos and Wang, 2001).

Since the Keap1-Nrf2-ARE pathway is closely related to the body’s resistance to oxidative stress, its use as a therapeutic target can achieve considerable efficacy while excessive activation of the pathway makes it difficult to avoid adverse effects and side effects. Although this pathway can be used in the treatment of cancer, over-activation of this pathway can, on the contrary, greatly increase the chance of cancer induction and, in addition, may cause diseases such as atherosclerosis (Gonzalez-Donquiles et al., 2017; Lignitto et al., 2019). Over-activation of Nrf2 can increase the survival advantage of tumors or even develop chemotherapy resistance, therefore, another idea for tumor treatment can be adopted, namely, targeted inhibition of Nrf2 and thus sensitization therapy. Besides, there are few natural compounds such as opium bitter alcohol and lignan that can be used as Nrf2 inhibitors in clinical practice. These would suggest that Nrf2 is a double-edged sword, and when conducting treatment, a reasonable individualized treatment plan should be designed and the correct dosing regimen should be chosen according to the patient’s condition and individual circumstances.

### MAPK Pathway

**FIGURE 5:**
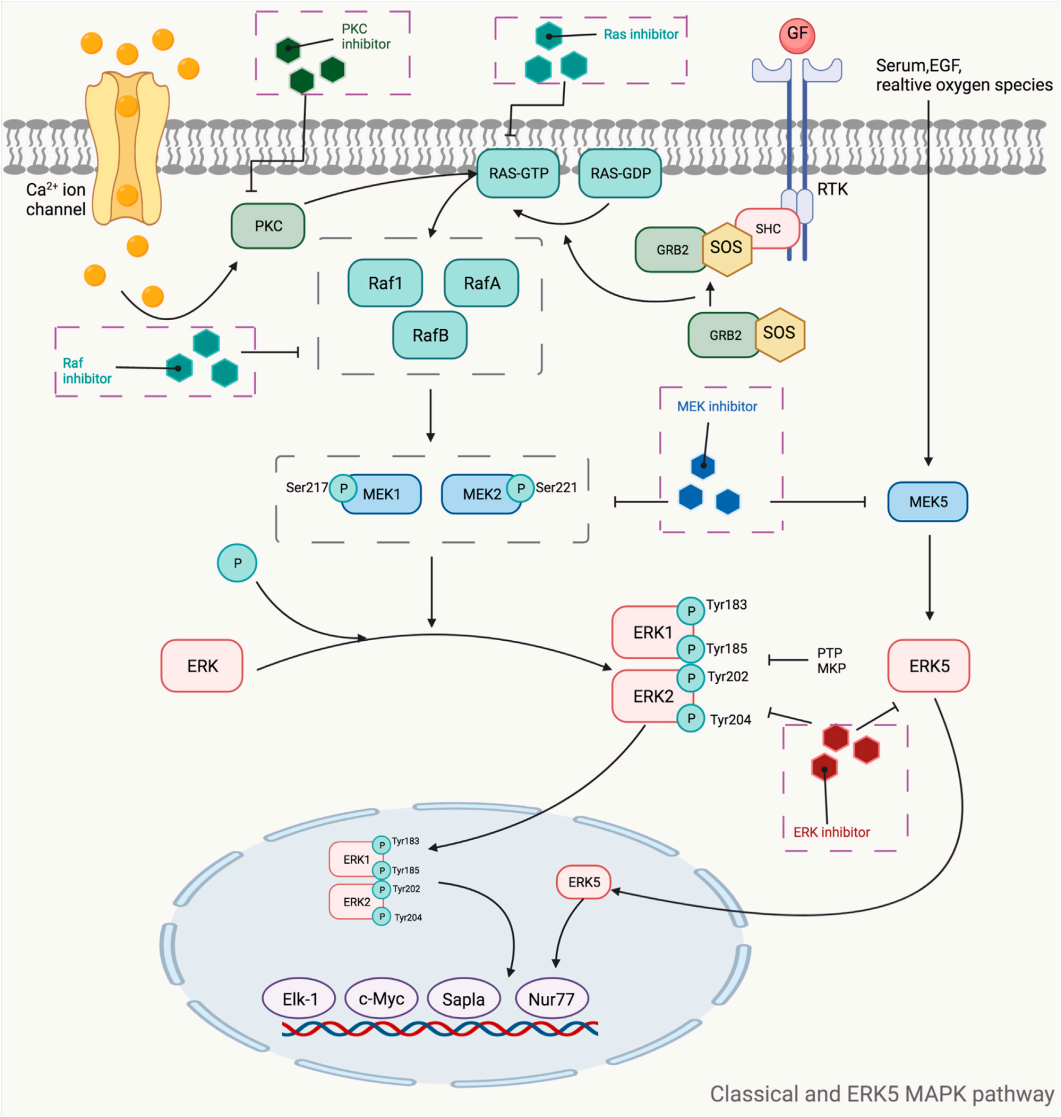
Mechanistic map of the classical and ERK5 MAPK signaling pathway. c-Myc, cell-myc; EGF, epidermal growth factor; Elk-1, ETS domain-containing protein Elk-1; ERK, extracellular regulated protein kinases; GDP, guanosine diphosphate; GTP, guanosinetriphosphate; GRB2, growth factor receptor-bound protein 2; MEK, mitogen-activated protein kinase kinase; MKP, mitogen-activated protein kinase phosphatase1; Nur77, nerve growthfactor-induced gene B; PKC, protein kinase C; PTP, protein tyrosine phosphatase; Ras, rennin angiotensin system; RTK, receptor tyrosine kinases; SHC, Src homology 2 domain containing; SOS, son of sevenless The figure is created with BioRender.com.

**FIGURE 6:**
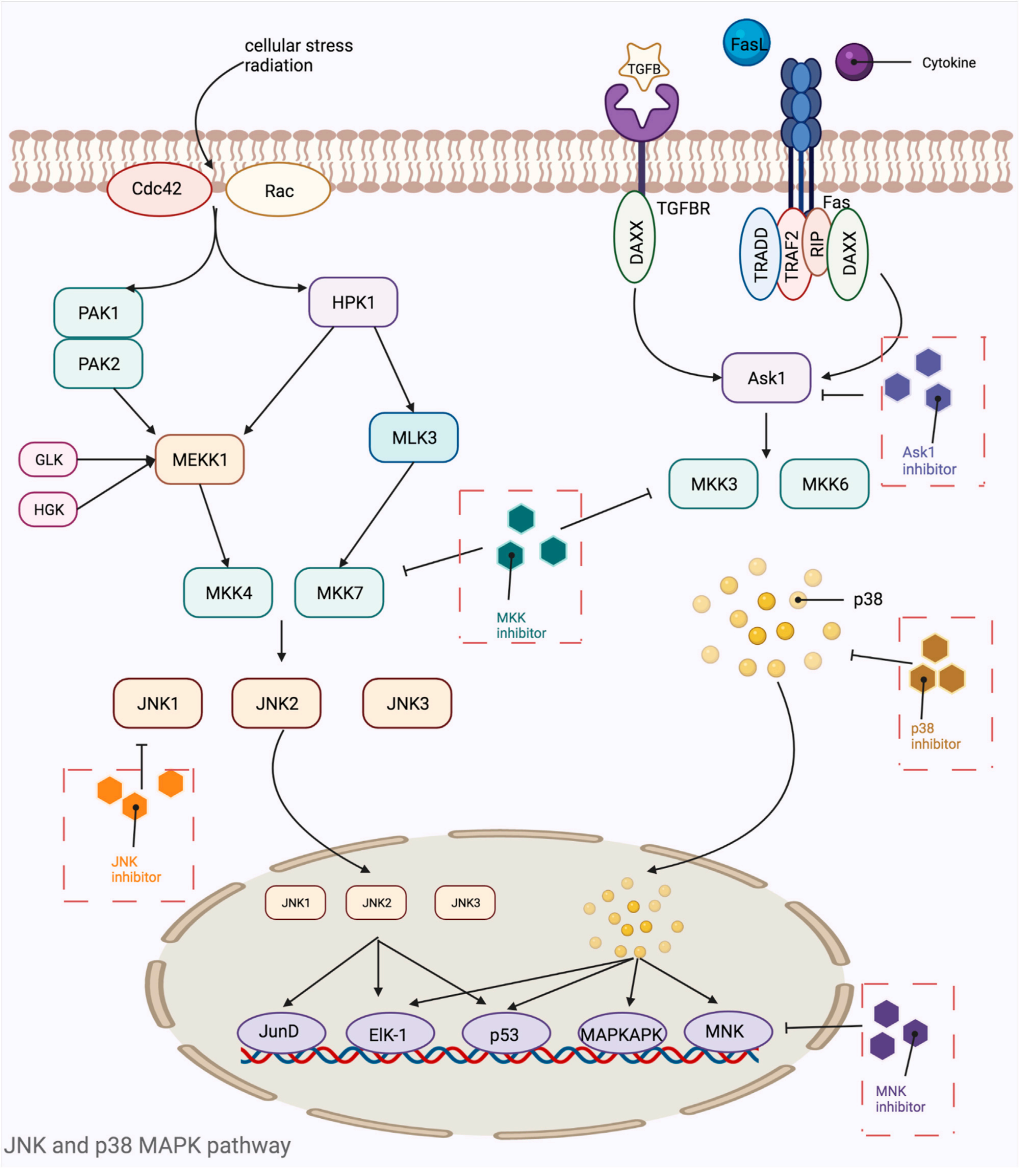
Mechanistic map of the JNK and p38 MAPK signaling pathway. Ask1, apoptosis signal-regulating kinase 1; Cdc42, cell division cycle 42; DAXX, death domain-associated protein; Elk-1, ETS domain-containing protein Elk-1; Fas, fatty acid synthase; GLK, glucokinase; HGK, human glandular kallikrein; HPK, histidine protein kinases; JNK, c-Jun N-terminal kinase; MAPKAPK, mitogen activated protein kinase activated protein kinase; MKK, mitogen-activated protein kinase kinase; MLK, mixed lineage kinase; MNK, mitogen-activated protein kinase interacting kinase; PAK, p21-activated kinase; Rac, Ras-related C3 botulinum substrate; RIP, receptor-interacting protein; TGFB, transforming growth factor; TRADD, TNF receptor-associated death domain; TRAF, tumor necrosis factor receptor associated factor. The figure is created with BioRender.com.

MAPKs are a class of threonine or serine protein kinases that are expressed in all eukaryotic cells. Activation of MAPK shows a typical three-stage enzymatic cascade reaction, namely MAP3K-MAP2K-MAPK chain. Upon activation of the upstream protein by cytokines, cellular stress, hormones, and neurotransmitters, the chain shows a cascade phosphorylation, thus transmitting the upstream signal to the downstream response molecules, which in turn are involved in the cellular anti-stress and anti-inflammatory responses. MAPKs signaling pathways play an important role in mediating cellular responses, and are widely involved in cell growth and reproduction, apoptosis, and a variety of cellular biochemical reactions. The pathways mediated by these four isoforms (JNK pathway, p38MAPK pathway, MEK5/ERK5 pathway, and ERK1/ERK2 pathway) are widely involved in the inflammatory, oxidative stress, and extracellular metabolic responses of cells in the body. ERK is one of the first MAPK isoforms to be identified and has five isoforms (ERK1 to ERK5), among which ERK1 and ERK2 are the most intensively studied and have a high degree of homology (Guo et al., 2008). The signaling pathway mediated by ERK1/ERK2 is mainly a signaling axis consisting of Ras, Raf, MEK, and ERK, through which upstream signals are transmitted step by step, leading to the regulation of multiple downstream genes, ultimately leading to the regulation of multiple genes downstream. In normal resting cells, Ras binds to GDP in an inactive state, while when the cell is stimulated by the outside world, Ras binds to GTP, which has one more phosphate group than GDP, and converts to an active state. The extra phosphate group puts the two switches (threonine-35 and glycine-60) in a “load spring” state, and when the phosphate group is released, the switch site shifts back to the inactive state (Santarpia et al., 2012). Ras-GTP induces Raf binding to Ras, mobilizes inactive proteins in the cytoplasm, and causes Raf kinase to accumulate at the cytosolic membrane (Chong et al., 2003; Wellbrock et al., 2004). When the Ras-Raf complex reaches the cell membrane, Ras can activate the function of the Raf isoform of serine/threonine kinase. When the Ras-Raf complex reaches the cell membrane, Ras activates the function of the Raf isoform of serine/threonine kinase. Activated Raf with its C-terminal catalytic region binds to MEK, phosphorylating the two serines (Ser221 and Ser217) in its subregion and activating MEK. Activated MEK can in turn phosphorylate the dual threonine and tyrosine sites on ERK, which activates ERK (Tyr183 and Tyr185 for ERK1 phosphorylation sites and Tyr202 and Tyr204 for ERK2 phosphorylation sites). ERK1 and ERK2 are activated for nuclear translocation and regulate many effector genes that will be relevant to cell proliferation, differentiation, survival, growth, and angiogenesis (Crews et al., 1992; Meloche and Pouysségur, 2007; Mebratu and Tesfaigzi, 2009). The ERK5 pathway, also known as the BMK1 pathway, can be activated by epidermal growth factors and a variety of extracellular stimuli, including hyperosmolarity, hypoxia, oxidants, and fluid shear stress. Tyr218 and Tyr220 on ERK5 are activated by regulation of the upstream protein kinase MAPKK5, which, like ERK1 and ERK2, undergoes nuclear translocation and regulates the corresponding genes regulation (Figure 5). The ERK5 pathway is also important for cell proliferation and differentiation and organogenesis. For example, Sohn et al. showed that it is ERK5, but not ERK1/2, that plays a key role in the developmental maturation of thymocytes, revealing that ERK5 has a role in mediating the differentiation of T lymphocytes (Sohn et al., 2008). The c-Jun amino-terminal transferase (JNK) was discovered during the study of a series of biological processes (UV responses) in cells exposed to ultraviolet radiation (UV), and it mainly regulates the phosphorylation of activated proteins such as c-Jun (Devary et al., 1992). The JNK pathway is activated after cells are exposed to various biotic or abiotic stress events, such as infection, inflammation, oxidative and other stresses, DNA damage, osmotic stress, or cytoskeletal changes (Zeke et al., 2016). In addition, G proteins such as Rac, CDC-42, tumor necrosis factor receptor-associated factor-based bridging proteins, and death-effector domain-containing proteins can also regulate JNK activation (Schattenberg et al., 2012). MAPK is activated by MAPK kinases (MKKs, MEKs, JNKKs, MAP2Ks), which are activated by MKK kinases (MEKKs, MAPKKKs, MAP3Ks). The first MAP3K found to activate JNK was MEKK1 (Minden et al., 1994). Subsequently, MEKK2 and MEKK3, MEKK4, mixed family kinases 2 and 3 (MLK2, MLK3), double leucine pull chain kinase (DLK), tumor transposon-2 (Tpl-2), TGF-β activating kinase (TAK1), apoptosis signaling regulatory proteases 1 and 2 (ASK1, ASK2), and 1001 amino acid kinases 1 and 2 (Tao1, Tao2) were identified (Karin and Gallagher, 2005). The two MAP2Ks specific to the JNK pathway are MKK4 and MKK7, with MKK4 more likely to phosphorylate the 185th tyrosine residue of JNK, while MKK7 prefers the 183rd tyrosine residue (Lawler et al., 1998). After JNK is activated, it then activates numerous downstream substrates that are involved in numerous intracellular functions, including apoptosis, cytoskeletal reorganization, transcriptional activity, and universal proteinization (Chen et al., 2001). The most common p38MAPK activators are lipopolysaccharides, in addition to osmotic stress, oxidative stress, UV exposure, heat shock, hypoxia, ischemia, interleukin-1β (IL-1β), tumor necrosis factor-α (TNF-α), and transforming growth factor-β (TGF-β), and neuropathic pain (Koul et al., 2013). Unlike the JNK pathway, p38 is mainly activated by two MAPKKs, MKK3 and MKK6, and activation of p38 requires simultaneous dual phosphorylation of threonine and tyrosine.As mentioned before G proteins such as Rac, CDC-42 can activate the JNK pathway, and its activation of p38 is also promoted. Rac1 can bind to MEKK1 or MLK1, while Cdc42 can only bind to MLK1, both of which can lead to the activation of p38 through MAP3Ks (Tibbles et al., 1996; Hirai et al., 1997). p38 then nuclear translocates and acts on downstream substrates, which include a large number of transcription factors such as activated transcription factors 1, 2 and 6 (ATF-1/2/6), SRF accessory protein (Sap1), CHOP (growth arrest and DNA damage inducible gene 153, or GADD153), p53, C/EBPβ, myocyte enhancer factor 2C (MEF2C) MEF2A, MITF1, DDIT3, ELK1, NFAT, and high mobility histone box protein 1 (HBP1) (Zarubin and Han, 2005) (Figure 6). Due to the complexity of the MAPK signaling pathway, resulting in a wealth of targets for its action, researchers have so far conducted a lot of research and development on inhibitors of various targets on this pathway. Table 5 shows a summary of some of the targeted inhibitors of the MAPK pathway, with a list of their indications and stages of research.

**TABLE 5:** Targets in and inhibitor targeting the MAPK pathway.

	Target	Agent	Phase	Indication	References
Ras inhibitor	Ras processing	Lonafarnib	approved	Hutchinson-Gilford Progeria Syndrome	Moore et al. (2020)
		Tipifarnib	investigational	colorectal cancer, Leukemia (myeloid), Pancreatic Cancer, Solid Tumors	Moore et al. (2020)
		Cysmethynil	experimental	Malignant Pleural Effusion, Neoplasm	Winter-Vann et al. (2005)
		Deltarasin	experimental	Adenocarcinoma, Neoplasm	Zimmermann et al. (2013)
		NHTD	experimental	Neoplasm	Leung et al. (2019)
		UCM-1336	experimental	Neoplasm	Marín-Ramos et al. (2019)
	KRAS-G12C	Sotorasib	approved	KRAS G12C mutant Non Small Cell Lung Cancer, Colorectal Cancer, Appendix Cancer	Canon et al. (2019)
		Adagrasib	investigational	KRAS G12C mutant Lung, Colon Adenocarcinomas	Hallin et al. (2020)
		JNJ-74699157	investigational	Advance Solid Tumor	Janes et al. (2018)
		LY3499446	investigational	Advance Solid Tumor	Uprety and Adjei, (2020)
		ARS-853	experimental	Neoplasm	Lito et al. (2016)
		ARS-1620	experimental	Neoplasm	Patricelli et al. (2016)
	SOS1-mediated nucleotide exchange on RAS	Sulindac	approved	Osteoarthritis, Rheumatoid Arthritis, Ankylosing Spondylitis, Acute Painful Shoulder, Acute Gouty Arthritis	O'Bryan Pharmacological targeting of RAS, (2019)
		BI-2852	experimental	Neoplasm	O'Bryan Pharmacological targeting of RAS, (2019)
		DCAI	experimental	Heart Disease	O'Bryan Pharmacological targeting of RAS, (2019)
		Kobe0065	experimental	Colonic Neoplasm, Colorectal Neoplasm	O'Bryan Pharmacological targeting of RAS, (2019)
	SOS	BAY-293	experimental	Neoplasm, Non-Small-Cell Lung Carcinoma	Hillig et al. (2019)
		BI-3406	experimental	Non-Small-Cell Lung Carcinoma	Karoulia et al. (2016)
		BI-1701963	investigational	Advanced and Metastatic Solid Tumor	Karoulia et al. (2016)
	SHP2	JAB-3068	investigational	Advance Solid Tumor	Karoulia et al. (2016)
		TNO155	investigational	Advance Solid Tumor	Karoulia et al. (2016)
		RMC-4550	experimental	Neoplasm, Neuroblastoma	Nichols et al. (2018)
Raf inhibitor	Raf	Dabrafenib	approved	Specific types of Melanoma, Non-Small Cell Lung Cancer, Thyroid Cancer	Karoulia et al. (2016)
		Encorafenib	approved	Unresectable or Metastatic Melanoma with specific mutations	Karoulia et al. (2016)
		Sorafenib	approved	Unresectable Liver Carcinoma, Advanced Renal Carcinoma	Karoulia et al. (2016)
		Vemurafenib	approved	Metastatic Melanoma	Karoulia et al. (2016)
		Belvarafenib	investigational	Neoplasm, Melanoma	Nichols et al. (2018); Kim, (2019)
		LXH-254	investigational	Neoplasm, Non-Small-Cell Lung Carcinoma	Nichols et al. (2018) Monaco, (2019)
		LY3009120	investigational	Neoplasm, Melanoma	Peng, (2015); (Vakana et al., 1202017)
		PLX8394	investigational	Advanced Unresectable Solid Tumors	Karoulia et al. (2016)
		AZ-628	experimental	Neoplasm, Melanoma	(Vakana et al., 1202017)
		TAK632	experimental	Systemic Inflammatory Response Syndrome, Melanoma, Neurodegenerative Diseases	Nakamura et al. (2013); Okaniwa et al. (2013)
MEK inhibitor	MEK1	Selumetinib	approved	Several types of Cancer	Lee and Duesbery, (2010)
		HL-085	investigational	Cancer	Tian et al. (2013)
		RO4987655	investigational	Neoplasm	Cheng and Tian, (2017)
		G-573	experimental	Neoplasm	Cheng and Tian, (2017)
		PD318088	experimental	Neoplasm	Cheng and Tian, (2017)
	MEK5	BIX02188	experimental	Neuralgia, Substance Withdrawal Syndrome	Drew et al. (2012)
		BIX02189	experimental	Cardiomegaly, Acute Myeloid Leukemia	Drew et al. (2012)
	MEK1/MEK2	Binimetinib	approved	Metastatic Melanoma with specific mutations	Pheneger et al. (2006)
		Cobimetinib	approved	Unresectable or Metastatic Melanoma	Cheng and Tian, (2017)
		Trametinib	approved	Specific types of Melanoma, Non-Small Cell Lung Cancer, Thyroid Cancer	Cheng and Tian, (2017)
		AZD-8330	investigational	Advance Solid Tumor	Wallace et al. (2009)
		CI-1040	investigational	Breast Cancer, Colorectal Cancer, Lung Cancer, Pancreatic Cancer	Barrett et al. (2008)
		GDC-0623	investigational	Metastatic Solid Tumors	Hatzivassiliou et al. (2013)
		PD-0325901	investigational	Melanoma, Solid Tumors, Advanced Cancer, Breast Neoplasm	Barrett et al. (2008)
		Pimasertib	investigational	N-Ras Mutated Locally Advanced or Metastasis Malignant Cutaneous Melanoma, Ovarian Cancer	Cheng and Tian, (2017)
		Refametinib	investigational	Hepatocellular Cancer, Melanoma, Colorectal Cancer	Iverson et al. (2009)
		TAK733	investigational	Advanced Non-Hematologic Malignancies Advanced Metastatic Melanoma	Dong et al. (2011)
		WX-554	investigational	Advance Solid Tumor	Jamieson et al. (2016)
		CInQ-03	experimental	Fibrosarcoma, Sarcoma, Neoplasm	Cheng and Tian, (2017)
		PD184161	experimental	Hepatocellular Carcinoma, Neoplasm	Cheng and Tian, (2017)
		PD98059	experimental	Hyperalgesia, Edema, Hypertrophy	Cheng and Tian, (2017)
		RO5068760	experimental	Neoplasm, Melanoma	Isshiki et al. (2011)
		SL327	experimental	Drug-Induced Dyskinesia, Cocaine-Related Disorders	Cheng and Tian, (2017)
	Raf/MEK1/MEK2	RO5126766	investigational	Neoplasm	Isshiki et al. (2011)
ERK inhibitor	ERK1/ERK2	CC-90003	investigational	Mesenteric Ischemia, Peripheral Nervous System Diseases	Kidger et al. (2018)
		KO-947	investigational	Non-Small Cell Lung Cancer	Kidger et al. (2018)
		LTT462	investigational	Unresectable or Metastatic Melanoma	Kidger et al. (2018)
		LY-3214996	investigational	Neoplasm, Melanoma	Kidger et al. (2018)
		MK-8353	investigational	Neoplasm, Melanoma, Chronic Brain Damage	Kidger et al. (2018)
		Ravoxertinib	investigational	Locally Advanced or Metastatic Solid Tumors	Kidger et al. (2018)
		Ulixertinib	investigational	Tumor	Kidger et al. (2018)
		DEL-22379	experimental	Neoplasm	Kidger et al. (2018)
		FR180204	experimental	Neoplasm	Kidger et al. (2018)
		Vtx-11e	experimental	Neoplasm, Retinoblastoma	Kidger et al. (2018)
	ERK5	XMD8-92	experimental	Neoplasm, Myeloid Leukemia	Drew et al. (2012)
p38 MAPK inhibitor	p38	ARRY371797	investigational	Dilated Cardiomyopathy	Banerjee et al. (2012)
		BIRB 796	investigational	Chemical and Drug Induced Liver Injury, Crohn Disease	Bagley et al. (2010)
		BMS582949	investigational	Rheumatoid Arthritis, Inflammation	Banerjee et al. (2012)
		Pamapimod	investigational	Osteoporosis, Rheumatoid Arthritis	Bagley et al. (2010)
		PF03715455	investigational	Chronic Obstructive Pulmonary Disease	Banerjee et al. (2012)
		PH797804	investigational	Pulmonary Disease, Chronic Obstructive	Banerjee et al. (2012)
		SB681323	investigational	Pulmonary Disease, Chronic Obstructive	Banerjee et al. (2012)
		VX745	investigational	Werner Syndrome, Rheumatoid Arthritis	Banerjee et al. (2012)
		SB203580	experimental	Cardiomyopathies, Chemical and Drug Induced Liver Injury	Banerjee et al. (2012)
		SB239063	experimental	Middle Cerebral Artery Infarction	Banerjee et al. (2012)
		SB706504	experimental	Chronic Obstructive Pulmonary Disease	Banerjee et al. (2012)
		SD0006	experimental	Arthritis, Rheumatoid Arthritis	Banerjee et al. (2012)
		RO3201195	experimental	Werner Syndrome	Bagley et al. (2010)
		UR-13756	experimental	Werner Syndrome	Bagley et al. (2010)
ASK1 inhibitor	ASK1	Selonsertib	investigational	Nonalcoholic Steatohepatitis, Bridging (F3) Fibrosis	(Rosenkranz et al., 2017; Schuster et al., 2017; Loomba et al., 2018; Younossi et al., 2018; Chertow et al., 2019; Ji et al., 2019)
		BPyO-34	experimental	Autoimmune Disorders, Cancer	Starosyla et al. (2015)
		GS-444217	experimental	Fibrosis, Glomerulonephritis, Inflammation	(Tesch et al., 2015; Amos et al., 2018; Budas et al., 2018; Liles et al., 2018)
		GS-459679	experimental	Liver Injury	He et al. (2016); Xie et al. (2015); Gerczuk et al. (2012)
		GS-627	experimental	Arthritis, Inflammation	Nygaard et al. (2018)
		MSC2032964A	experimental	Neurodegenerative Diseases, Cardiovascular Diseases	Guo et al. (2010)
		TC ASK 10	experimental	Chronic Obstructive Pulmonary Disease	Eapen et al. (2018); Terao et al. (2012)
JNK inhibitor	JNK1	AV-7	experimental	Diabetes	Yao et al. (2009)
		Isoquinolone derivatives	experimental	Heart Failure	Asano et al. (2008)
		PYC71N	experimental	Hyperosmotic Stress	Haynes et al. (2012); Ngoei et al. (2011)
		PYC98	experimental	Hyperosmotic Stress	Haynes et al. (2012); Ngoei et al. (2011)
	JNK3	Brimapitide	investigational	Infarction, Nerve Degeneration	Beydoun et al. (2015); Desir et al. (2018)
		Pyridopyrimidinone derivatives	experimental	No Data	Zhao et al. (2012)
		Quinazoline	experimental	Edema, Malaria, Hyperalgesia	He et al. (2011)
		Triazolothione 1	experimental	CNS Diseases	Neitz et al. (2011)
		6-anilinoindazoles	experimental	No Data	Swahn et al. (2005)
		20-anilino-4,40-bipyridines	experimental	No Data	Swahn et al. (2006)
	JNK1/JNK2	4-quinolone analogues	experimental	Asthma	Gong et al. (2012)
	JNK1/JNK3	4-fluorophenyl isoxazoles	experimental	No Data	He et al. (2014)
	JNK1/JNK2/JNK3	Bentamapimod	investigational	Endometriosis	Okada et al. (2016)
		CC-401	investigational	Myeloid Leukemia	Wu et al. (2020b)
		CC-930	investigational	Acute Kidney Injury, Fibrosis	Plantevin Krenitsky et al. (2012)
		AS601245	experimental	Acute Monocytic Leukemia	(Cerbone et al., 20122012); Cerbone et al. (2012)
		BI-78D3	experimental	Diabetes Mellitus, Inflammation, Insulin Resistance	Posthumadeboer et al. (2012); Stebbins et al. (2008)
		Ginsenoside Rg1	experimental	Candidiasis, Cardiovascular Diseases	Zhang et al. (2015)
		JNK-IN-1	experimental	Anticancer Potential for Skin Cancer, Attenuation of Chronic Colitis	Kersting et al. (2013); Gao et al. (2009)
		JNK-IN-8	experimental	Sensitizes Triple-negative Breast Cancer Cells to lapatinib	Ebelt et al. (2017)
		Pyrazolanthrone	experimental	Acute Lung Injury, Asthma	Cicenas et al. (2017)
MNK inhibitor	MNK	Cercosporamide	experimental	Neoplasm	Hou et al. (2012)
		CGP052088	experimental	Neoplasm	Hou et al. (2012)
		CGP57380	experimental	Giloma, Neoplasm	Hou et al. (2012)
MLK inhibitor	MLK	CEP1347	investigational	Nerve Degeneration, Noise-Induced Hearing Loss	Handley et al. (2007)
		CEP11004	experimental	Nerve Degeneration	Handley et al. (2007)
		K252a	experimental	Pheochromocytoma, Depressive Disorder	Handley et al. (2007)
PKC inhibitor	PKC	Isopropyl myristate	approved	Allergic Contact Dermatitis	Haarberg et al. (2013)
		Enzastaurin	investigational	Brain Cancer, Lymphoma (non-hodgkin), Lung Cancer	Son et al. (2011)
		Ruboxistaurin	investigational	Type 2 Diabetes Mellitus, Type 1 Diabetes Mellitus	Son et al. (2011)
		Sotrastaurin	investigational	Uveal Melanoma, Richter Syndrome, Prolymphocytic Leukemia, Recurrent Mantle Cell Lymphoma, Recurrent Small Lymphocytic Lymphoma	Skvara et al. (2008)
		Calphostin C	experimental	Whooping Cough, Neoplasm	Son et al. (2011)
		Chelerythrine	experimental	Hyperalgesia, Stomach Ulcer	Son et al. (2011)
		GF 109203X	experimental	Edema, Hyperalgesia	Son et al. (2011)
		Rottlerin	experimental	Acute kidney injury, Fever	Son et al. (2011)
		Ro 31–8220	experimental	Whooping Cough	Son et al. (2011)
		Staurosporine	experimental	Chromosome-Defective Micronuclei, Edema	Son et al. (2011)

Sulindac is an FDA-approved drug for the treatment of autoimmune diseases such as rheumatoid arthritis that blocks the MAPK pathway and whose primary target is HRAs (one of four highly homologous proteins encoded by the Ras gene) (O'Bryan Pharmacological targeting of RAS, 2019). Subsequently, it was shown that sulforaphane sulfide, a metabolite of sulforaphane, could directly block Ras activation of Raf and reduce Ras-mediated transformation *in vitro* (Herrmann et al., 1998). Of course, sulforaphane does not only act on one pathway, but it can also exert its effects by inhibiting the NF-κB pathway, which has been shown to inhibit NF-κB activation by binding to the ATP-binding site of IKK and regulating RelA nuclear translocation (Berman et al., 2002). Approximately 93% of sulindac and the prototype drug and 98% of the sulfated metabolites are bound to serum albumin after oral administration, with the liver being an important elimination pathway. Currently, approximately 50% of sulindac is excreted in the urine, and studies have found that sulindac can be excreted from rat milk, while it is debatable whether it can be excreted from human milk. Between the complexity of Ras targeting and the fact that the Ras family is one of the most commonly mutated genes in tumor cancers, researchers have put a great deal of effort into the family. Until a decade ago, researchers had not found an effective Ras inhibitor, so much so that Ras was used as an ineffective therapeutic target. However, after nearly 3 decades of research, a breakthrough point in Ras inhibitor research has emerged and tremendous progress has been made to date, with tremendous scope for research. The first Ras inhibitor to enter clinical trials was AMG510, which has now been cleared by the FDA for marketing. It was found that the target of action is mainly KRAS-G12C, which can be covalently inhibited by cysteine at codon 12 of the gene, whereas wild-type KRAS does not have covalently bindable cysteine specifically, so AMG510 is a specific target drug for this commonly mutated region of G12C (Moore et al., 2020). Subsequently, researchers have identified other Ras inhibitors that are partially in clinical trials, such as Adagrasib, JNJ-74699157, and LY3499446, while some are still in the preclinical study evaluation stage and have not entered clinical trials or marketing, such as ARS-853 and ARS-1620. In addition to the target KRAS-G12C, Shokat et al. identified and defined for the first time a metamorphic binding pocket located in the switch-II region of the G12C mutation, for which they designed a series of irreversible inhibitory compounds that resulted in a good inhibitory effect on the pathway (Ostrem et al., 2013), thus showing a new research direction and a good research prospect for Ras inhibitors.

Although the treatment of cancer patients or patients with neurodegenerative lesions with inhibitors targeting this pathway has shown a good trend of prolonged survival and good improvement of lesion symptoms, these inhibitors still inevitably cause side effects. For example, Stephnie et al. reported that patients treated with the MEK inhibitor trametinib for melanoma experienced prolonged visual loss that did not completely resolve after discontinuation of the drug and could even progress to extensive uveitis and multiple plasmacytoid retinal detachments (Sarny et al., 2017). The mechanism of the complication of this ocular side effect cannot be elucidated at this time, but physicians and pharmacists are cautioned to use the medication carefully and to adjust it within the therapeutic window. In addition to this, in a study of the patient population when trametinib was combined with dabrafenib in the treatment of non-small cell lung cancer, it was found that most patients experienced systemic adverse effects such as fever, skin inflammation, mouth ulcers, diarrhea, and loss of appetite (Chalmers et al., 2019), but this was mainly less related to the inhibition of the MAPK pathway and more due to drug metabolites or drug. This is less related to MAPK pathway inhibition and more to pathological changes caused by drug metabolites or drugs themselves. However, this is also a warning to physicians and pharmacists to master the balance between the therapeutic effects of drugs and adverse drug reactions.

## Conclusion

This article reviews representative targets and their inhibitors on the JAK-STAT, NF-κB, PI3K-AKT-mTOR, MAPK, and Keap1-Nrf2-ARE pathways, and indicates their current research stages and indications, thus facilitating researchers to conduct in-depth comparative studies on drugs with the same targets. A large number of studies and clinical observations have demonstrated the efficacy of targeted immunosuppressive agents in chronic inflammatory diseases, but a variety of adverse effects or ethical issues have resulted in relatively few marketed drugs for human use, and most of the drugs found to be effective have been forced to end up in clinical trials or preclinical studies. Researchers are now working to find commonalities between immunosuppressive agents of the same target and to study the structural similarities of the drugs, thus facilitating further development work on the target molecules. In addition to this, researchers face the challenge of studying the targeting of drugs to specific cells or tissues, i.e. the detailed study of the pharmacokinetics of a particular drug. It is hoped that the therapeutic potential and safety of small molecule immunosuppressive agents/targeted therapy immunosuppressive agents will be further demonstrated and evaluated to achieve more interventions, improvements and treatments for chronic diseases.

Although the widespread use of immunosuppressive agents has solved many problems in autoimmune diseases and organ transplantation, among others, the road to immunosuppression has never stopped. Currently, the world is still facing a shortage of donor organs for transplantation, which will mean that we will need to find xenogeneic donors, such as pigs, thus alleviating the lack of donors. For example, today porcine xenografts and hepatocyte transplants are gradually being classified into human treatment options for liver diseases. However, it has been found that xenografted porcine grafts can cause many adverse reactions such as rejection, coagulation disorders, and thrombocytopenia while performing a liver support role (Li et al., 2022). Even with porcine modified donor livers (PERV-KO/3-KO/9-TG), humoral rejection, interstitial hemorrhage, and inflammatory injury still occur. Therefore, the focus of transplantation is now more towards porcine allogeneic hepatocyte transplantation. The lower immunogenicity of genetically modified porcine hepatocytes has led to a much higher success rate of transplantation, and it is hypothesized that the key to successful cellular xenotransplantation is related to the source of blood for liver perfusion. In contrast to whole organs that are perfused by the donor’s vessels, the blood supply for cellular grafts originates from the recipient (Parker et al., 1996; Cascalho and Platt, 2001). Although the success rate of surgery is gradually improving, there is still a need to pursue more efficient immunosuppression combined with more excellent genetic modification protocols, which can not only solve the problem from the donor, but also improve the prognostic quality of the recipient in the postoperative period.This raises thoughts and requirements for in-depth development and flexible combination applications of immunosuppressive agents.
